# The innate immunity of guinea pigs against highly pathogenic avian influenza virus infection

**DOI:** 10.18632/oncotarget.16503

**Published:** 2017-03-23

**Authors:** Kun Zhang, Wei wei Xu, Zhaowei Zhang, Jing liu, Jing Li, Lijuan Sun, Weiyang Sun, Peirong Jiao, Xiaoyu Sang, Zhiguang Ren, Zhijun Yu, Yuanguo Li, Na Feng, Tiecheng Wang, Hualei Wang, Songtao Yang, Yongkun Zhao, Xuemei Zhang, Peter R. Wilker, WenJun Liu, Ming Liao, Hualan Chen, Yuwei Gao, Xianzhu Xia

**Affiliations:** ^1^ Key Laboratory of Jilin Province for Zoonosis Prevention and Control, The Military Veterinary Institute, Academy of Military Medical Science of PLA, Changchun, 130122, PR China; ^2^ Philips Institute for Oral Health Research, School of Dentistry, Virginia Commonwealth University, Richmond, Virginia, 23298, USA; ^3^ CAS Key Laboratory of Pathogenic Microbiology and Immunology, Institute of Microbiology, Chinese Academy of Sciences, Beijing, 100101, PR China; ^4^ Department of Influenza Vaccine, Changchun Institute of Biological Product, Changchun, 130062, PR China; ^5^ College of Veterinary Medicine, South China Agricultural University, Guangzhou, 510642, PR China; ^6^ Department of Microbiology, University of Wisconsin La Crosse, La Crosse, Wisconsin, 54601, USA; ^7^ State Key Laboratory of Veterinary Biotechnology, Harbin Veterinary Research Institute, Chinese Academy of Agricultural Sciences, Harbin, 150001, PR China

**Keywords:** innate immunity, guinea pig, highly pathogenic avian influenza virus, GBP-1, RIG-I

## Abstract

H5N1 avian influenza viruses are a major pandemic concern. In contrast to the highly virulent phenotype of H5N1 in humans and many animal models, guinea pigs do not typically display signs of severe disease in response to H5N1 virus infection. Here, proteomic and transcriptional profiling were applied to identify host factors that account for the observed attenuation of A/Tiger/Harbin/01/2002 (H5N1) virulence in guinea pigs. RIG-I and numerous interferon stimulated genes were among host proteins with altered expression in guinea pig lungs during H5N1 infection. Overexpression of RIG-I or the RIG-I adaptor protein MAVS in guinea pig cell lines inhibited H5N1 replication. Endogenous GBP-1 expression was required for RIG-I mediated inhibition of viral replication upstream of the activity of MAVS. Furthermore, we show that guinea pig complement is involved in viral clearance, the regulation of inflammation, and cellular apoptosis during influenza virus infection of guinea pigs. This work uncovers features of the guinea pig innate immune response to influenza that may render guinea pigs resistant to highly pathogenic influenza viruses.

## INTRODUCTION

Influenza A viruses pose a continual threat to human health in the form of seasonal epidemics and occasional pandemics [1]. Seasonal epidemics occur as a result of the ongoing mutational and selective processes among influenza viruses circulating through the human population, which give rise to antigenically distinct viral variants that circumvent preexisting immunity. In contrast, pandemic viruses can emerge as a result of genomic reassortment between different influenza subtypes and the spillover of influenza viruses from animals to humans [2].

The first human H5N1 infections were identified in Hong Kong in 1997. Since 2003, H5N1 viruses have circulated widely, infecting hundreds of people in 16 countries with a fatality rate approaching 60% [3]. Despite widespread circulation of H5N1 viruses in wild birds and domestic poultry, relatively few human cases have occurred and there is little evidence of human to human transmission. Recent research has demonstrated that a limited number of mutations can render H5 viruses transmissible by respiratory droplets in mammals, further heightening concerns that H5N1 viruses may serve as a source of the next human pandemic [4–6].

Host defense against influenza A virus infection is initiated by the innate immune system. Innate immune responses are initiated when molecular patterns, such as distinctive features of influenza virus RNA, are recognized by host cell expressed pattern recognition receptors (PPRs) [7, 8]. PPR sensing of viral infection triggers the production of pro-inflammatory cytokines and chemokines and the induction of adaptive immune responses [9]. In addition, the complement system acts as an important part of the innate immune response, and has recently been implicated in immune defense against influenza viruses [10].

Overabundant inflammatory immune responses correlate with increased morbidity and mortality during infection with influenza viruses, including highly pathogenic H5N1 viruses and the pandemic 2009 H1N1 influenza virus [11, 12]. A ‘cytokine storm’, or excessive production of inflammatory cytokines, is a consistent feature of infection of both humans and experimental animals with H5N1 viruses and is widely thought to be a key component of viral pathogenesis culminating in extensive pulmonary edema, acute bronchopneumonia and alveolar hemorrhage [13, 14]. The molecular mechanisms that dictate the balance between protective and pathological immune responses against different strains of influenza virus remain unclear.

Animal models have been widely used to study influenza virus infection and host immune responses [15, 16]. Inbred, transgenic, and knockout mice have served as an invaluable resource for studies aiming to uncover the molecular and cellular processes underpinning both protective and pathogenic host responses to influenza virus infection. Ferrets, which are widely regarded as a preferred small animal model of influenza virus infection, are commonly used to evaluate the virulence and transmission dynamics of influenza viruses. Although more resource intensive, rhesus macaques have also been used to model human infection with influenza viruses [17, 18]. Guinea pigs represent an alternative animal model for the study of influenza viruses. Guinea pigs are susceptible to infection with human influenza virus isolates, are relatively inexpensive when compared to ferrets, and can be used to study the viral characteristics that affect respiratory droplet mediated transmission.

Avian H5N1 influenza and the ‘Spanish’ influenza virus of 1918 exhibit high levels of virulence in mice, ferrets, and macaques, but fail to induce significant morbidity or mortality in guinea pigs despite efficient virus replication in the upper respiratory tract [19–22]. The apparent disparity in influenza induced pathology in guinea pigs as compared to other well-established animal models offers an opportunity to uncover the protective host responses that limit influenza virulence. With the recent completion of the *Cavia porcellus* sequencing project, proteomic and genetic analyses of the guinea pig response to influenza infection are possible [23].

Proteomic analysis of host cellular responses to virus infection can be used to identify potential cellular factors involved directly or indirectly in viral infection. Mass spectrometry (MS)-based approaches identify and quantify thousands of proteins from cellular samples at different time points following viral infection [24–26]. Quantitative proteomics has been applied to study host cell responses to influenza infection, and previous work has primarily focused on infection of human cell lines [27–29].

Isobaric tags for relative and absolute quantification (iTRAQ) in combination with multidimensional liquid chromatography and tandem MS (LC-MS/MS) analysis is emerging as a powerful platform to identify disease specific targets. Here, we have used iTRAQ and LC-MS/MS along with transcriptional analyses to provide a global view of the host response during H5N1 influenza virus infection of guinea pigs. H5N1 infection of guinea pigs was found to induce expression of numerous proteins with diverse functions in the innate immune system. However, excessive pro-inflammatory cytokines production, which is a hallmark of H5N1 infection in other animal models, was notably absent during H5N1 infection of guinea pigs. Several differentially expressed genes identified through proteomic and transcriptional analyses, including RIG-I, MAVS, Mx1, and complement protein C3, were shown to limit viral replication in guinea pig cell lines or in guinea pigs. These results suggest a number of candidate genes that may contribute to the observed resilience of guinea pigs to highly pathogenic avian influenza viruses.

## RESULTS

### H5N1 influenza virus replicates in guinea pigs but does not elicit pathology

**Figure 1:**
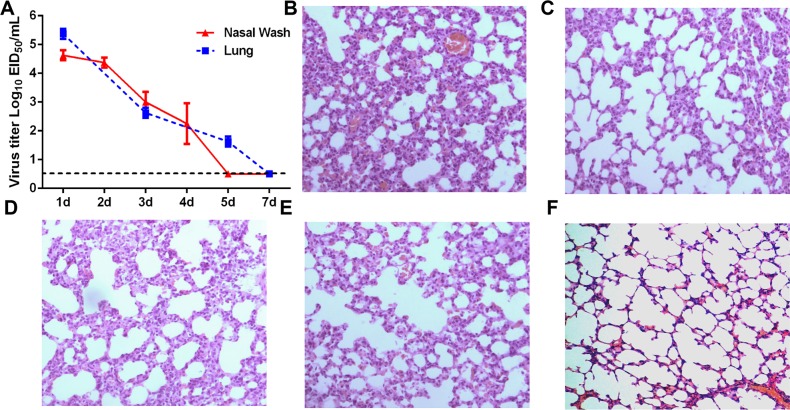
Virus titers and histological analysis of guinea pigs infected with H5N1 **(A)** Guinea pigs were infected with H5N1 virus and viral titers measured in lung tissue and nasal washes at the indicated time points. **(B-F)** Hematoxylin and eosin stained lung sections prepared from samples harvested from H5N1-infected guinea pigs on day 1 **(panel B)**, day 3 **(panel C)**, day 5 **(panel D)** and day 7 **(panel E)** post-infection or from guinea pigs mock-infected with PBS as a control **(panel F)**.

Guinea pigs were intranasally inoculated with 10^5^ EID_50_ of H5N1 virus and were assessed for weight loss, nasal wash and lung viral titers, and lung pathology. As expected, inoculated guinea pigs did not display overt clinical signs or show significant reductions in body weight when compared to mock inoculated animals (data not shown). Virus titers in nasal washes and lung tissue progressively declined following a peak 1 day post infection (dpi) (Figure 1A). Virus levels were below the limit of detection in nasal washes and lungs by 5 and 7 dpi, respectively (Figure 1A). Mild alveolar wall thickening and an accumulation of macrophages and neutrophils were noted in the lungs of infected guinea pigs on 1, 3 and 5 dpi, although these histological changes were less severe at 3 and 5 dpi (Figure 1B, 1C and 1D). Although guinea pigs still had slight pulmonary inflammation at 7 dpi, the presence of inflammatory cells infiltration was negligible (Figure 1E).

### Host defense protein response profile

**Figure 2:**
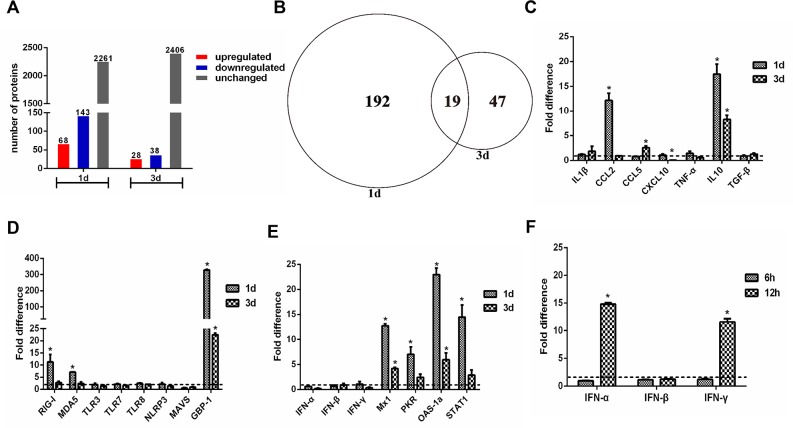
Quantitative proteome and gene expression analysis of H5N1 infected guinea pig lungs **(A)** Enumeration of differentially expressed proteins in the lungs of H5N1 infected guinea pigs as compared to uninfected controls. Differentially expressed proteins are defined by detection at ≥1.5 or ≤0.67 the levels found in uninfected controls. **(B)** Venn diagram showing the distribution of differential expressed proteins indicated in **panel A**. **(C-F)** qRT-PCR analysis of expression of cytokine and chemokine genes **(panel C)**, pattern recognition receptor related genes **(panel D)**, and interferon response associated genes **(panel E, F)** in H5N1 infected guinea pig lungs relative to uninfected controls. The fold difference was determined using the 2^−ΔΔCt^ method and RNA levels were normalized to β-actin. The data represent the means of three independent experiments. Error bars represent standard deviation.

Protein extracts were prepared from H5N1-infected and mock-infected guinea pig lungs 1 and 3 dpi. A total of 2472 proteins were detected by iTRAQ coupled 2D LC-MS/MS analysis. When individual protein levels from H5N1-infected guinea pig lung extracts were compared to mock-infected guinea pig control extracts, 258 proteins displayed significantly altered expression. Sixty-eight and 28 proteins were increased at 1 and 3 dpi, respectively, whereas 143 and 38 proteins were decreased at 1 and 3 dpi, respectively. Notably, fewer proteins displayed altered patterns of expression were observed at 3 dpi as compared to 1 dpi (Figure 2A and 2B). A ≥1.5- or ≤0.67-fold difference in protein level and a two-tailed p-value <0.05 between H5N1-infected and mock infected extracts at each time point was considered significant. The false discovery rate (FDR) of the sample sets was 1.2%, indicating very high reliability of the proteins identified. Western blot analyses performed on a selected set of proteins confirmed iTRAQ quantification results (Supplementary Figure 1). Proteins with significantly altered expression levels between H5N1- and mock-infected guinea pigs were classified into four clusters: (1) proteins that were increased at 1 dpi and/or 3 dpi, (2) proteins that were decreased at 1 dpi and/or 3 dpi, (3) proteins increased at 1 dpi and decreased at 3 dpi, and (4) proteins decreased at 1 dpi and increased at 3 dpi (Supplementary Table 1).

Twenty-two of the differentially expressed proteins have known immune defense functions (Supplementary Table 1, bold italic letters). Among these, indoleamine 2,3-dioxygenase 1-like (IDO-1) and interferon-induced GTP-binding protein Mx2-like (Mx2) were increased while aminoacyl tRNA synthase complex interacting multifunctional protein 1 was decreased at 1 and 3 dpi. Annexin A1-like, lysozyme C-like, complement C3 preproprotein and lactotransferrin-like were increased at 1 dpi whereas pulmonary surfactant-associated protein D-like (SPD) was decreased at 1 dpi. Differential expression of two S100 calcium binding proteins (protein S100-A8-like and protein S100-A9-like) was noted. S100 A8 was increased at 1 dpi and 3 dpi and S100 A9 was increased at 1 dpi and then decreased at 3 dpi. In addition, signal transducer and activator of transcription 1-like (STAT1) and mitochondrial antiviral-signaling protein-like (MAVS) were increased and decreased at 1 dpi and 3 dpi, respectively. The 40S ribosomal protein S15a-like and proteasome subunit alpha type 2-like were increased at 1 dpi, while stathmin-like, barrier to autointegration factor-like and SPARC-like protein 1-like were decreased.

Gene ontology (GO) enrichment analysis was performed to identify GO terms that were over- or underrepresented among the 258 differentially expressed proteins identified by iTRAQ coupled 2D LC-MS/MS. Among the set of differentially expressed proteins identified at 1 dpi, GO enrichment analysis identified a significant enrichment (P < 0.05) in 120 biological process terms, 13 cell component terms, and 9 molecular function terms (Supplementary Table 2). The three most common GO term categories at 1 dpi were defense response, response to biotic stimulus, and response to other organism (Supplementary Figure 2). At 3 dpi, the most common GO term categories were camera type eye development, positive regulation of cytokine production and defense response (Supplementary Figure 2). GO term statistical analysis revealed 20 genes and 8 genes involved in defense response at 1 and 3 dpi, respectively. The immune system response, regulation of leukocyte and lymphocyte mediate immunity were also enriched at 1 dpi by GO analysis (Supplementary Table 2). These observations reveal an induction of innate immune response phase by 1 dpi which declined by 3 dpi in guinea pigs.

### Transcriptional profiling of guinea pig lung tissue following H5N1 infection

Proinflammatory cytokine production has been closely associated with morbidity and mortality following H5N1 infection in humans, mice, ferret, and nonhuman primates [22, 30]. However, proteomic analyses did not reveal significant increases in the production of proinflammatory cytokines in guinea pigs infected with H5N1 on days 1 and 3 post infection. To confirm these proteomic results at the transcriptional level, a panel of genes with known host defense and antiviral activity were analyzed by real time RT-PCR (Supplementary Table 3). Expression of the cytokines IL-1β, CCL5, CXCL10, TNF-α, and TGF-β was not significantly altered in the lungs of guinea pigs infected with H5N1 when compared to mock-infected controls (Figure 2C). In contrast, CCL2 expression was significantly increased at 1 dpi and IL-10 expression was significantly increased at both 1 and 3 dpi (Figure 2C). Among PRRs, TLR3, TLR7, TLR8, and NLRP3 did not display significantly altered mRNA levels in guinea pig lungs at 1 or 3 dpi whereas RIG-I and MDA5 were markedly increased 1 dpi (Figure 2D). Consistent with changes identified through proteomic analysis, GBP-1 mRNA was increased and MAVS mRNA decreased in H5N1 infected guinea pigs when compared to mock infected controls (Figure 2D).

The interferon response-related genes Mx1, STAT1, OAS-1a, and PKR were increased in the lungs of guinea pigs infected with H5N1 at 1 and 3 dpi when compared to mock-infected controls (Figure 2E). While expression of IFN-inducible proteins including Mx2, IDO-1 and GBP-1 were detected following AIV infection via proteomic analysis, IFN expression was not detected by LC-MS/MS at either 1 or 3 dpi. Consistent with the proteomic data, IFN-α, IFN-β and IFN-γ transcript levels in H5N1 infected guinea pig lungs were similar to levels in the lungs of mock-infected animals at both 1 and 3 dpi (Figure 2E). At 6 and 12 hours post infection, however, IFN α and IFN γ mRNA levels increased by approximately 10- and 20-fold, respectively, when compared to mRNA levels in mock-infected lungs (Figure 2F). Collectively, these results show that H5N1 infection of guinea pigs results in robust early expression of IFNs and interferon-stimulated genes but does not elicit the excessive production of pro-inflammatory cytokines associated with poor outcomes in humans and other animal models following H5N1 infection.

### Functional analysis of differentially expressed genes on H5N1 replication in guinea pig cell lines

**Figure 3:**
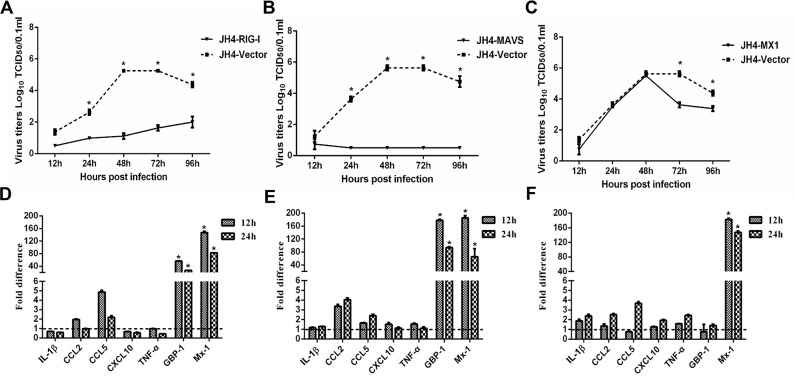
Functional analysis of RIG-I, MAVS and Mx1 in H5N1 infected guinea pig cells JH4 cells were transfected with an expression vector to overexpress RIG-I **(panels A and D)**, MAVS **(panels B and E)**, or Mx1 **(panels C and F)**. Overexpression of vector served as a control. Forty eight hours after transfection, cells were infected with H5N1 influenza virus at the MOI of 0.01. Culture supernatants were collected at the indicated time points to determine viral titers by TCID_50_ **(panels A and C)**. Cells were harvested at the indicated time points and cellular RNA extracted to analyze a select panel of immune related genes by quantitative RT-PCR **(panels D and F)**. RNA levels were normalized to β-actin. Fold differences were determined using the 2^−ΔΔCt^ method as compared to vector control overexpressing cells. The data represent the means of three independent experiments. Error bars represent standard deviation.

The proteomic and transcriptional profiles of H5N1 infected guinea pigs lungs identified a number of differentially expressed proteins and genes that may contribute to reduced H5N1 virulence in guinea pigs as compared to other mammalian animal models. To directly explore whether select differentially expressed genes limit H5N1 viral replication *in vitro*, we transiently overexpressed RIG-I, MAVS, Mx1, STAT1, GBP-1, SPD, HSP90 and IDO-1 in JH4 guinea pig fetal lung fibroblasts and measured H5N1 replication kinetics. Overexpression of the pattern recognition receptor RIG-I or the adaptor protein MAVS in JH4 fibroblasts resulted in significantly reduced viral titers when compared to control cells at each time point tested (Figure 3A and 3B). Similarly, overexpression of Mx1 significantly reduced viral titers in JH4 cells at 72 hours post infection, but did not affect viral replication during the first 48 hours (Figure 3C). Overexpression of STAT1, GBP-1, SPD, HSP90 and IDO-1 did not lead to significant reductions in viral titers when compared to vector control expressing control cells (Supplementary Figure 3). Similar results were obtained in experiments using 104C1 guinea pig embryonic fibroblasts (data not shown). These results show that overexpression of RIG-I, MAVS and Mx1 restricts H5N1 replication in guinea pig cell lines.

We next explored the impact of RIG-I, MAVS and Mx1 overexpression on the expression of innate immune response genes during infection of JH4 guinea pig fetal lung fibroblasts. RIG-I and MAVS overexpression resulted in marked increase of Mx1 and GBP-1 at both 12 and 24 hours post-infection when compared to H5N1-infected control cells (Figure 3D and 3E). CCL2 and CCL5 expression was increased to a greater extent in H5N1 infected JH4 cells overexpressing either RIG-I or MAVS as compared to H5N1 infected control cells, whereas minimal changes in expression were noted for IL-1β, CXCL10, and TNF-α (Figure 3D and 3E). Transient overexpression of Mx1 resulted in significantly increased levels of Mx1 mRNA as expected, but did not increase GBP-1 mRNA levels (Figure 3F). Overexpression of RIG-I or MAVS also induced expression of Mx1 and GBP-1 in uninfected JH4 cells, suggesting that transcriptional activation of Mx1 and GBP-1 in response to RIG-I or MAVS was not dependent on H5N1 infection (Supplementary Figure 5). Consistent with the observation that STAT1, GBP-1, SPD, HSP90 and IDO-1 overexpression did not affect viral replication in JH4 cells, overexpression of these proteins failed to induce expression of Mx1 and other innate immune response genes, including CCL2, CXCL10, and TNF-α (Supplementary Figure 4). Taken together, these results suggest that RIG-I and MAVS overexpression may limit viral replication in part due to induction of Mx1 and GBP-1.

### The RIG-I mediated antiviral response is dependent on GBP-1

**Figure 4:**
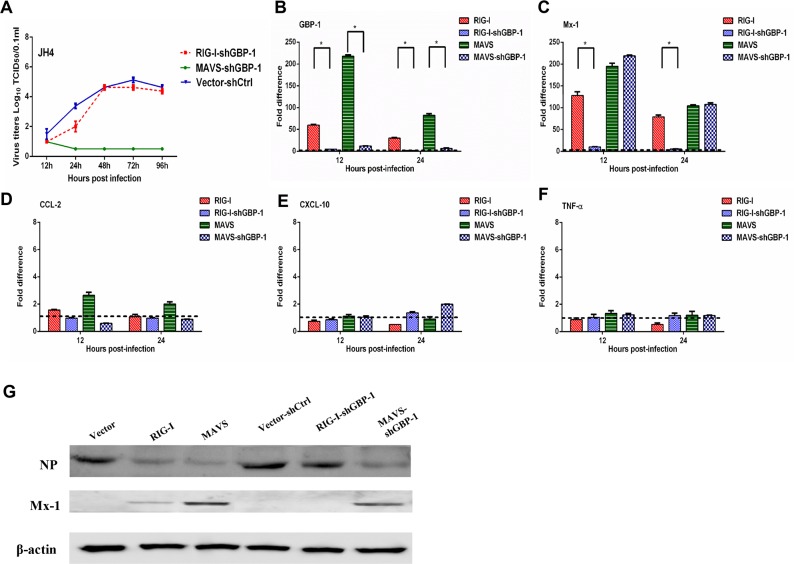
GBP 1 is required for RIG I mediated inhibition of H5N1 replication in guinea pig JH4 cells JH4 cells were transfected with an expression plasmid to overexpress RIG-I, MAVS, or control vector and either a shRNA targeting GBP-1 (shGBP-1) or a shRNA control (shCtrl). Forty eight hours after transfection, cells were infected with H5N1 virus at an MOI of 0.01. Culture supernatants were collected at the indicated time points post infection to determine virus titer by TCID_50_ **(panel A)**. RNA harvested from cells was used to evaluate expression of the indicated genes by quantitative RT-PCR **(panels B-F)**. RNA levels were normalized to β-actin. The fold difference from the qRT-PCR was determined using the 2^−ΔΔCt^ method and are expressed as fold induction as compared to cells overexpressing RIG-I or MAVS co-transfected with the shRNA control. The data represent the means of three independent experiments. Error bars represent standard deviation. At 16 h post infection, cells were harvested and analyzed by Western blot to assess NP and Mx1 protein levels. β-actin served as a loading control **(Panel G)**.

GBP-1 has been identified as an IFN-inducible protein that inhibits influenza virus replication in cell lines [31, 32]. As shown above, overexpression of RIG-I or MAVS in guinea pig JH4 cells restricted H5N1 viral replication and significantly increased GBP- 1 expression following infection (Figure 3). We therefore asked whether the antiviral effects of RIG-I and MAVS were dependent upon the GBP-1 induction following H5N1 infection. Expression of endogenous GBP-1 was knocked down using a shRNA specific for guinea pig GBP-1 mRNA in JH4 cells transiently overexpressing MAVS, RIG-I, or an empty vector as a control. Whereas RIG-I overexpression in JH4 cells strongly inhibited H5N1 replication (Figure 3A), overexpression of RIG-I with concurrent GBP-1 knockdown abrogated the RIG-I mediated inhibition of viral replication (Figure 4A). In contrast, overexpression of MAVS with concurrent GBP-1 knockdown did not affect the ability of MAVS to inhibit viral replication, indicating that MAVS restricts H5N1 replication independently of GBP-1 or functions downstream of GBP-1 (Figure 4A).

As expected, expression of GBP-1 was vastly reduced in H5N1 infected JH4 cells overexpressing RIG-I and MAVS with concurrent GBP-1 knockdown at both 12 and 24 hours post infection relative to cells overexpressing RIG-I and MAVS without GBP-1 knockdown (Figure 4B). GBP-1 knockdown in cells overexpressing RIG-I resulted in significantly reduced induction of Mx1 mRNA at 12 and 24 hours post infection, whereas Mx1 expression in H5N1 infected cells overexpressing MAVS was not affected by GBP-1 knockdown (Figure 4C). Similar results were obtained in 104C1 cells, where GBP-1 knockdown nearly abolished the ability of overexpressed RIG-I to limit viral replication and promote transcription of Mx1 (Supplementary Figure 6A, 6C). In contrast, GBP-1 knockdown in JH4 and 104C1 cells overexpressing RIG-I or MAVS did not significantly alter expression of CCL2, CXCL10 or TNF-α mRNA (Figure 4D–4F and Supplementary Figure 6D-6F).

Consistent with the above results showing restricted H5N1 replication in JH4 cells overexpressing RIG-I or MAVS, H5N1 infected JH4 cells overexpressing RIG-I or MAVS displayed lower levels of influenza NP protein when compared to H5N1 infected JH4 cells transfected with an empty vector as a control (Figure 4G). Concurrent knockdown of GBP-1 in H5N1 infected JH4 cells overexpressing RIG-I displayed NP levels comparable to H5N1-infected control cells, further indicating a requirement for GBP-1 in RIG-I mediated inhibition of H5N1 replication (Figure 4G). GBP-1 knockdown in MAVS-overexpressing JH4 cells did not impact NP levels following H5N1 infection relative to NP levels seen in MAVS-overexpressing JH4 cells without GBP-1 knockdown (Figure 4G). While overexpression of RIG-I increased Mx1 protein levels in H5N1 infected JH4 cells relative to control cells, concurrent GBP-1 knockdown abolished Mx1 protein expression (Figure 4G). These data show that GBP-1 is required for RIG-I-mediated restriction of H5N1 replication in guinea pig cells.

### Effect of complement on pathogenicity and the regulation of inflammation following H5N1 virus infection

**Figure 5:**
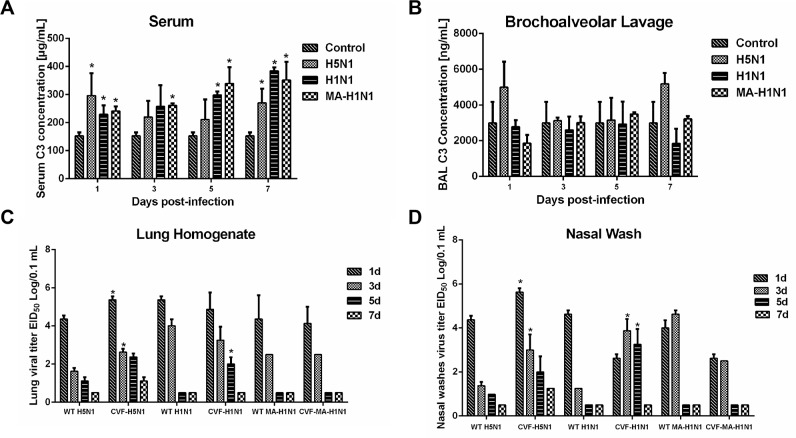
Complement component C3 contributes to H5N1 and H1N1 clearance in guinea pigs **(A-B)**, Guinea pigs were infected with H5N1, H1N1, or a mouse adapted H1N1 virus (MA H1N1) and C3 concentrations in the serum **(panel A)** and bronchoalveolar lavage **(panel B)** measured at the indicated time points. Guinea pigs mock-inoculated with PBS served as a control. Error bars represent standard deviation. **(C-D)**, Guinea pigs were infected with H5N1, H1N1, or MA H1N1 virus following treatment with CVF to deplete C3 activity or PBS as a control. Lung homogenates **(panel C)** and nasal washes **(panel D)** were collected at the indicated time points to determine virus titer by EID_50_ analysis. The data represent the means of three independent experiments. Error bars represent standard deviation. The asterisks indicate a significant difference (p < 0.05).

To validate our approach for identifying functio-nally relevant host genes, we additionally characterized expression levels of the C3 component of complement which was identified as an induced protein in the lungs of H5N1-infected guinea pigs at 1 dpi when compared to mock-infected controls. Infection of guinea pigs with A/Tiger/Harbin/01/2002 (H5N1) (HAB01), 2009 pandemic H1N1 A/Changchun/01/2009 (CH01) or a mouse-adapted A/Changchun/01/2009 H1N1 variant (MA CH01) resulted in increased concentrations of C3 in the serum when compared to uninfected controls (Figure 5A). In contrast, C3 levels were slightly increased in bronchoalveolar lavage collected from H5N1 infected guinea pigs at 1 and 7 dpi when compared to guinea pigs infected with either H1N1 virus (Figure 5B).

Increased complement has been associated with enhanced inflammation and tissue destruction. Thus, we hypothesized that the elevated complement levels in H5N1 infected guinea pigs could be involved in the generation of inflammatory responses during infection. Cobra venom factor (CVF) is a structural and functional analog of the C3 protein. Due to its stability, resistance to regulation, and fluid phase activity, CVF injection has been used to experimentally deplete complement activity. Following treatment with CVF to deplete complement or a mock treatment control, guinea pigs were intranasally inoculated with H5N1 (HAB01), H1N1 (CH01), or a mouse-adapted H1N1 virus (MA CH01). Guinea pigs depleted of complement activity by CVF treatment showed significantly increased H5N1 viral titers in the lungs and nasal washes at 1 and 3 dpi when compared to non CVF treated controls (Figure 5C and 5D). Depletion of complement activity similarly resulted in significantly higher virus titers in the lungs of H1N1 infected guinea pigs 5 dpi and in nasal washes at 3 and 5 dpi, whereas no differences in viral titers were noted when CVF treated guinea pigs were infected with mouse-adapted H1N1 (Figure 5C and 5D).

**Figure 6:**
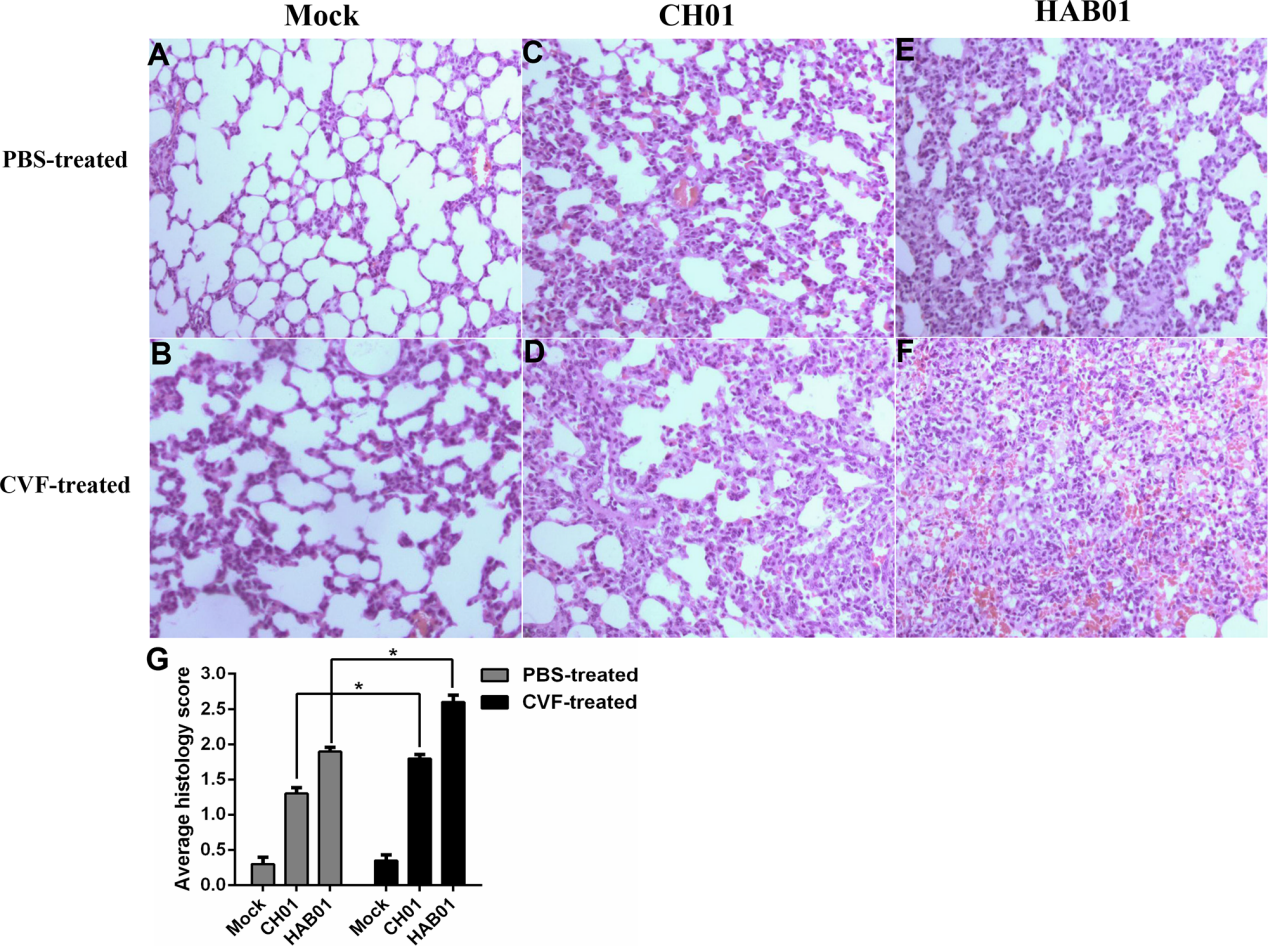
Complement depletion increases lung damage and cellular infiltration in H5N1 infected guinea pigs **(A-F)**, CVF or PBS treated guinea pigs were infected with H5N1 (HAB01), H1N1 (CH01), or were mock infected with PBS as a control. One day post infection, lung tissue was collected, formalin fixed, paraffin embedded, and stained with hematoxylin and eosin for histological analysis. Representative images are shown at 20× magnification. **G**, Mean histological scoring of H&E-stained lung sections shown in panel A-F (n=2 or 3, *P<0.05).

Lung samples were harvested from CVF-treated or untreated guinea pigs following infection with H5N1 or H1N1 virus to assess histological changes resulting from infection. CVF-treated guinea pigs were noted to have more severe pulmonary damage after infection with H5N1 or H1N1 influenza virus, presenting with moderate to severe thickening of alveolar walls and prominent infiltration of neutrophils, lymphocytes and macrophages (Figure 6). Overall, these data suggest that heightened levels of C3 in bronchoalveolar lavage fluid during H5N1 infection of guinea pigs may contribute to host defense by promoting viral clearance and limiting influenza induced lung pathology.

**Figure 7:**
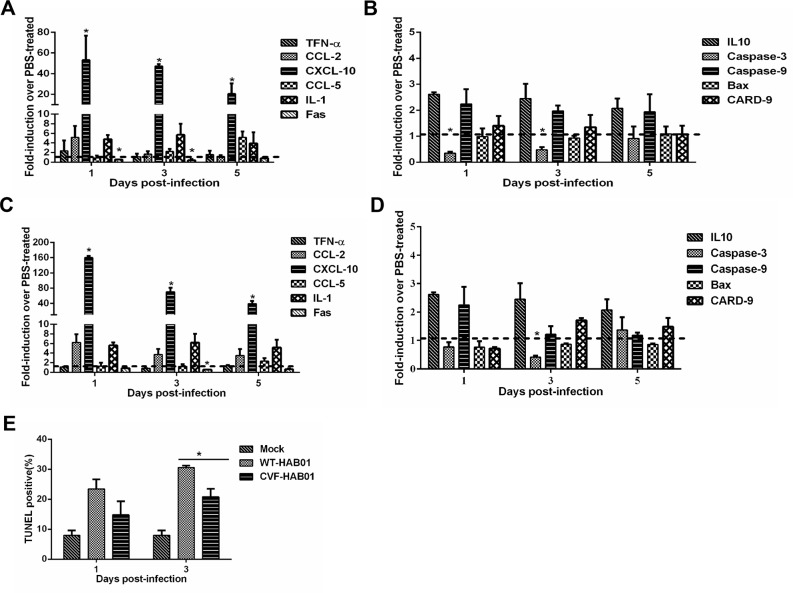
Complement depletion increases lung inflammation and decreases apoptosis following H5N1 and H1N1 infection of guinea pigs Guinea pigs were treated with CVF to deplete complement activity or PBS as a control. Twelve hours later, guinea pigs were infected with HAB01 H5N1 **(panels A and B)** or CH01 H1N1 virus **(panels C and D)**. Total RNA was harvested from lungs at the indicated time points post infection and analyzed for expression of genes associated with the inflammatory response **(panels A and C)** and cellular apoptosis **(panels B and D)** by quantitative RT PCR. RNA levels were normalized to β-actin. Fold induction was determined using the 2^−ΔΔCt^ method and are expressed as fold induction as compared to samples from PBS treated infected guinea pigs. The data represent the means of three independent experiments. Error bars represent standard deviation. **(E)**, Guinea pigs were treated with CVF or PBS as a control and then infected with HAB01 H5N1 virus. Lungs were collected at the indicated time points and assessed using a TUNEL assay. The percentage of TUNEL positive cells are shown (n=3). *P<0.05. Results are representative of 2-3 independent experiments.

Lung tissues were harvested from CVF-treated and PBS-treated guinea pigs at 1, 3 and 5 dpi to characterize expression of a panel of innate immune response and apoptosis related genes. Modest increases in the expression of CCL2, CCL5, and IL-1 were noted in CVF-treated guinea pigs infected with H5N1 (Figure 7A) or H1N1 (Figure 7C) when compared to PBS-treated guinea pigs infected with the respective virus. Strikingly, CXCL10 mRNA levels were significantly higher during infection with both H5N1 and H1N1 in guinea pigs depleted of complement via CVF treatment when compared to controls (Figure 7A and 7C). These data further support an important role for complement activation in the regulation of immune cell chemotaxis and inflammatory responses during influenza virus infection in guinea pigs.

Among apoptosis related genes, Fas and caspase-3 expression were significantly reduced during H5N1 and H1N1 infection of CVF-treated guinea pigs (Figure 7B and 7D), suggesting that complement activity may promote cellular apoptosis during infection. Terminal deoxynucleotidyl transferase dUTP nick end labeling (TUNEL) analysis was performed to quantify apoptotic cells in the lungs of guinea pigs infected with H5N1 with or without prior treatment with CVF. H5N1 infection of guinea pigs resulted in a substantial increase in TUNEL positive lung cells at both 1 and 3 dpi when compared to uninfected controls (Figure 7E). Prior treatment with CVF to deplete complement resulted in a reduction in the percentage of TUNEL positive cells compared to untreated H5N1 infected guinea pigs, although this difference was significant only at 3 dpi (Figure 7E). These data underscore a role for complement activation in the induction of cellular apoptosis during influenza virus infection.

## DISCUSSION

Highly pathogenic H5N1 avian influenza viruses cause severe disease in humans and commonly applied animal models including mice, ferrets, and rhesus macaques. In contrast, guinea pigs are reported to be highly resistant to H5N1 induced pathology as compared to other animal models [17, 18, 21]. Consistent with previous reports, guinea pigs did not present with obvious clinical signs following infection with H5N1 virus and developed only mild to moderate pulmonary lesions. Further, virus titers in guinea pig lungs and nasal washes rapidly declined following a peak at 1 dpi. The reduced virulence of H5N1 in guinea pigs presented an opportunity to identify host factors and immunological pathways that contribute to resistance against highly pathogenic influenza virus infection.

While numerous studies have documented the role of various influenza virus proteins in immune evasion, the factors that dictate the balance between protective and pathological host responses to infection are less well understood [2, 33] [18, 34]. The application of genome wide profiling techniques such as microarray analysis has resulted in the identification of novel host factors involved with H5N1 influenza virus infection of mice, ferrets and macaques [35–38]. The host response to H5N1 influenza infection has also been studied using proteomic approaches in macaques [39, 40]. In this study, we applied the iTRAQ approach to identify differentially expressed host proteins during infection in guinea pigs. Two hundred fifty-eight proteins displayed significantly altered expression levels at different time points post-infection. The majority of differentially expressed proteins were identified at 1 dpi, corresponding to peak virus titers in the lungs and nasal washes of infected guinea pigs. These results identify a number of cellular targets that may contribute to the guinea pig response to influenza virus infection.

The IFN-mediated immune response is associated with the transcriptional activation of hundreds of IFN-stimulated genes (ISGs) which collectively contribute to viral defense [41]. Consistent with this paradigm, we detected the increase of many IFN-inducible proteins, including Mx2, GBP-1, IDO-1 and STAT1, by LC-MS/MS at 1 and 3 dpi. While a number ISGs were identified at the protein level, IFN proteins themselves were not enriched on 1 or 3 dpi. At the level of transcription, robust induction of ISGs was noted at 1 and 3 dpi without significant increases in type I and II IFN mRNA, prompting an evaluation of IFN mRNA levels at earlier time points. IFN-α and IFN-γ mRNA levels were significantly induced at 12 hours post infection, whereas no evidence of IFN-β induction was noted. These results are in contrast with results obtained from mice and macaques in which IFN-β was the major type I IFN induced after influenza virus infection, suggesting that IFN-α may play a more important role in inducing immune response in the guinea pig during AIV infection [42, 43]. In addition, influenza virus NS1 protein can interact with human RIG-I to suppress the induction of IFN-β [44, 45]. Using immunoprecipitation assays, we confirmed an interaction of the influenza NS1 protein with guinea pig RIG-I (Supplementary Figure 7), suggesting IFN-β induction may be blocked by the interaction of NS1 with RIG-I in guinea pig cells. Apart from ISGs, a number of additional differentially expressed proteins with documented immune function were identified in the lungs of H5N1 infected guinea pigs. These proteins included S100 family proteins (S100 A8 and S100 A9), Annexin A1, SPD and CD44, which are involved in the regulation of the inflammasome response and inhibition of viral replication [27, 46].

Heat shock proteins (HSPs) are a class of multifunctional proteins that maintain cell stability when cells are exposed to elevated temperatures, pathogens and/or other environmental stresses. In the present study, we identified the increase of HSP90 at 1 dpi. Previous reports have documented a role of HSP90 in the nuclear import and assembly of the trimeric influenza polymerase complex [47]. Furthermore, a pharmacological HSP90 inhibitor inhibits influenza virus replication in cell culture [48]. The induction of HSP90 in guinea pig lungs during H5N1 infection may indicate that HSP90 contributes to host defense during viral infection, though HSP90 overexpression in guinea pig cell lines did not restrict H5N1 replication directly.

Intense inflammatory responses have consistently been reported as features of H5N1 pathogenesis in humans. NLRP3 and IL-1β, key components of the inflammasome, are activated in response to influenza virus infection resulting in the secretion of proinflammatory cytokines [49–52]. The early and sustained activation of NLRP3 and IL-1β production contributes to the infiltration of neutrophils and monocytes to sites of infection where these cells continue to secrete cytokines resulting in a “cytokine storm”. In the present study, proteomic and transcriptional analyses did not reveal induction of NLRP3 and IL-1β expression after infection of guinea pigs with H5N1 virus. There was also a lack of induction of other cytokines and chemokines commonly associated with severe H5N1 infection in mammals, including TNF-α, CCL5 and CXCL10 (IP10). These data suggest that the attenuated induction of proinflammatory immune mediators may limit H5N1-induced pathology in guinea pigs without compromising pathways that limit viral replication.

Although proteomic analyses failed to identify increases in PRR protein levels in the lungs of H5N1 infected guinea pigs, expression of both RIG-I and MDA5 were markedly increased at the transcriptional level. Consistent with the documented role of RIG-I in mice, RIG-I-mediated recognition of H5N1 plays a critical role in antiviral host defense in guinea pigs. Overexpression of RIG-I or the RIG-I adaptor protein MAVS in guinea pig cells strongly inhibited H5N1 replication and resulted in marked enhancement in the expression of Mx1 and GBP-1. The IFN-induced Mx1 protein has been shown to have potent antiviral activity against influenza viruses in humans and mice, although overexpression of Mx1 alone in guinea pig cells reduced viral titers *in vitro* only at 72 hours post infection. Importantly, the ability of RIG-I overexpression to restrict H5N1 replication *in vitro* was dependent upon GBP-1. These results uncover a previously unrecognized role for GBP-1 in the antiviral activities mediated by RIG-I upstream of the MAVS adaptor protein.

Several reports have shown that MAVS is required for RLR mediated immune responses. MAVS-deficient mice display impaired RIG-I- and MDA5-mediated interferon responses and are highly susceptible to RNA virus infection [53–55]. Given the documented role of MAVS in immune defense against RNA viruses, we explored the functional capacity of MAVS in guinea pig cells during H5N1 infection despite the observation that MAVS was decreased at the protein and transcriptional level in the lungs of H5N1-infected guinea pigs. Consistent with previous work, overexpression MAVS in guinea pig cells strongly inhibited H5N1 replication *in vitro*. Furthermore, overexpression of MAVS induced the expression of RIG-I, indicating a positive feedback network of MAVS in the RIG-I/MAVS signaling pathway. Several cellular and viral proteins are involved in the degradation of MAVS [56–58]. We speculate that reduced MAVS expression during H5N1 infection of guinea pigs may limit inflammation and resultant immunopathology without negatively impacting virus handling.

The complement system serves as a key component of the innate immune response and plays protective roles at mucosal surfaces. C3 deficiency in humans correlates with recurrent infections of the upper and lower respiratory tract [59]. Recent work has also demonstrated the protective role for complement C3 and hemolytic complement in H5N1 influenza pathogenesis in mice [37, 60]. Similar to what has been reported in mice, infection of guinea pigs with H1N1 and H5N1 influenza viruses caused significant increases in serum C3 levels. Depletion of C3 via CVF treatment delayed H5N1 and H1N1 influenza virus clearance in guinea pigs and was associated with increased lung damage during infection. Similar to previous reports, depletion of C3 in guinea pigs was associated with altered inflammatory cell infiltration to the lungs in response to influenza infection [60]. Interestingly, previous research has also shown that inhibition of complement activation in mice can limit lung injury and improve survival following H5N1 infection [61]. In addition, depletion of C3 suppressed influenza virus induced lung cell apoptosis in guinea pigs. Several reports have shown a relationship between complement activation and cellular apoptosis, including both anti- and pro-apoptotic effects of the complement system. Complement activity may act as a double-edged sword by limiting viral spread and contributing to influenza-induced lung injury [60, 62–66]. The mechanisms by which complement components influence cellular apoptosis and the inflammatory response during influenza virus infection in guinea pigs need to be further explored.

## MATERIALS AND METHODS

### Ethics statement

Study protocols were conducted in accordance with guidelines for animal welfare of the World Organization for Animal Health. All experimental protocols were approved by the Review Board Military Veterinary Research Institute of the Academy of Military Medical Sciences (Number SYXK2015 021). Studies with H5N1 AIVs were conducted in a biosecurity level 3 laboratory approved by the Military Veterinary Research Institute of the Academy of Military Medical Sciences.

### Cell lines and virus preparation

JH4 guinea pig fetal lung fibroblast cells (CCL-158) and 104C1 guinea pig embryonic fibroblast cells (CRL-1405) were obtained from ATCC and cultured according to the recommendations of the provider. Madin Darby canine kidney (MDCK) cells were obtained from ATCC and maintained in Dulbecco’s modified Eagle’s medium (DMEM) supplemented with 10% fetal bovine serum and 1% penicillin-streptomycin.

Highly pathogenic H5N1 avian influenza virus A/Tiger/Harbin/01/2002 (abbreviated HAB01), 2009 pandemic H1N1 A/Changchun/01/2009 (abbreviated CH01) and a mouse-adapted version of A/Changchun /01/2009 (abbreviated MA CH01) were obtained from the Military Veterinary Research Institute of the Academy of Military Medical Sciences. All viruses were grown in the allantoic cavities of 10 day-old specific pathogen free (SPF) embryonated chicken eggs for 72h at 37°C. Virus titers were determined by calculating the 50% egg infectious dose (EID_50_).

### Guinea pig studies

Specific pathogen- and viral antibody-free female Hartley strain guinea pigs (300–350g) were obtained from Beijing Merial Vital Laboratory Animal Technology Co. Ltd. (Beijing, China). Intramuscular injection of ketamine (20 mg/kg) and xylazine (1 mg/kg) were used to anesthetize animals.

For proteomic experiments, guinea pigs were anesthetized and inoculated intranasally (i.n.) with 300 μL phosphate buffered saline (PBS) or test virus (150 μL per nostril). Lungs were collected from three guinea pigs at each time point post-inoculation for analyses.

To deplete guinea pig of complement, 50 μg of cobra venom factor (CVF, Kunming Biogen Science & Technology Co. Ltd.) was injected intravenously. Control guinea pigs were injected with PBS only. Serum and brochioalveolar lavage (BAL) fluid was collected from euthanized guinea pigs 0.5, 2, 4, 6, 8 days after treatment for analysis of C3 levels by ELISA.

Twelve hours after injection with CVF or PBS, guinea pigs were anesthetized and inoculated intranasally (i.n.) with PBS or test virus in a 300 μL volume (150 μL per nostril). Nasal washes, blood, and lungs were collected from three animals in each group at different time points post-inoculation. BAL of the large lung lobe was performed with MEM containing 2% lidocaine. BAL fluid was centrifuged at 800 g for 10 minutes. The supernatant was collected and stored at -70°C for future analysis. A separate large lung lobe was washed and immediately stored in 10% neutralized phosphate buffered formalin for histological analysis. The remaining lobes and nasal washes were used for virus titration in eggs by EID_50_. After blood collection, serum was obtained by centrifugation, and stored at -30°C until analysis.

### Host defense and antiviral gene expression analysis by qRT-PCR

Total RNA was extracted from guinea pig lungs following inoculation using the RNeasy Mini kit (Qiagen). RNA (1 μg) was reverse transcribed to cDNA using the Superscript II reverse transcriptase kit (Invitrogen). qPCR was performed using Brilliant SYBR Green qPCR master mix (Agilent Technology) with specific primers (Supplementary Table 3). The relative expression of mRNA was normalized to β-actin expression using the comparative Ct method.

For proteomic analysis, lung homogenates were prepared from H5N1 infected and mock infected control lungs at 1 and 3 dpi. Samples were labeled with iTRAQ reagent Multiplex kit (AB Sciex) according to the manufacturer’s protocol. After labeling, the samples were pooled, dried and dissolved in SCX buffer A. Labeled peptides were fractionated by strong cation exchange chromatography (SCX) using Phenomenex Luna SCX. Each SCX fraction containing labeled peptides was analyzed twice by LC-MS/MS using an AB SCIEX Triple TOF 5600 mass spectrometer (AB Sciex) and online HPLC system (LC 20AD nanoHPLC, Shimadzu). MS data were acquired automatically in the positive ion mode, with a selected mass range of 300 2000 m/z. Peptides with +2 to +4 charge states were selected for MS/MS. Smart information dependent acquisition (IDA) was activated with automatic collision energy and automatic MS/MS accumulation. The fragment intensity multiplier was set to 20, and the maximum accumulation time was 2s. The Triple TOF 5600 mass spectrometer used for peptide identification has high mass accuracy (less than 2 ppm).

### Data analysis

Protein identification and relative quantitation was performed using Mascot 2.3.02 software (Matrix Science). Data files from both technical replicates of an iTRAQ sample set were processed together. The NCBI *Cavia porcellus* sequence database was used for protein identification. For protein abundance ratios measured using iTRAQ, we set a 1.5 fold change as the threshold and a two-tailed p value <0.05 to identify significant changes. False discovery rates were calculated using a concatenated normal and reversed sequence database and a previously reported method [67]. Gene Ontology (GO) functional classifications were analyzed with Blast2GO software and GO enrichment analysis was performed to identify GO terms that were significantly enriched in differentially expressed proteins. Clustering was done based on the relative quantitation results from the iTRAQ experiments.

### Western blot validation

Western blot analyses were performed to confirm differentially expressed proteins identified using the proteomic approach. Aliquots of the lung samples (20 μg protein) were separated by SDS PAGE and transferred onto PVDF membranes using a semi-dry transfer apparatus (Bio Rad). The PVDF membrane was incubated with SuperBlock Blocking Buffer (Pierce, Rockford, IL) containing 0.05% Tween 20 for 1 h, and then incubated with the indicated primary antibodies. Following incubation with the appropriate secondary antibody, SuperSignal West Dura Extended Duration Substrate (Pierce, Rockford, IL) was added. Images were obtained using a luminescent imaging system (LAS 4000; Fujifilm, Tokyo, Japan). Band intensity was quantified using the software Multi Gauge 3.1 (Fujifilm). The primary antibodies used in this study were reactive with Mx1, Fas-L, STAT1, HSP-70, HSP-90, IDO-1, BAX, Caspase-9, SPD (Santa Cruz Biotechnology Inc.), MAVS and β-actin (Abcam).

### Plasmid construction and cell transfection

Full length cDNA encoding guinea pig RIG-I, MAVS and Mx1 were amplified from guinea pig lung RNA using reverse transcription polymerase chain reaction (RT PCR). For mammalian cell expression, the RIG-I, MAVS and Mx1 coding sequences were subcloned into the pIRES2-EGFP vector (Invitrogen, Carlsbad, CA, USA) to produce the pIRES2-EGFP-RIG-I, pIRES2-EGFP-MAVS and pIRES2-EGFP-Mx1 plasmids for overexpression studies. The unmodified pIRES2-EGFP plasmid served as a control. To transiently reduce GBP-1 expression, a shRNA targeting GBP-1 was designed and synthesized by GenePharma Co., Ltd (Shanghai, China) and cloned into a plasmid to generate the pGPU6/GFP/Neo shGBP-1 knockdown vector. The pGPU6/GFP/Neo sh NC (shNC) vector targets a sequence not found within the guinea pig sequence database and was used as a control.

For overexpression studies, JH4 and 104C1 cells were transiently transfected with the pIRES2-EGFP-RIG-I, pIRES2-EGFP-MAVS, pIRES2-EGFP-Mx1, or empty pIRES2-EGFP expression plasmids using Lipofectamine 2000 (Invitrogen). In experiments to assess the impact of GBP-1 knockdown, cells were co-transfected with a protein expression plasmid and pGPU6/GFP/Neo shGBP-1 or pGPU6/GFP/Neo sh NC. At 48 h post-transfection, cells were infected with HAB01 at a multiplicity of infection (MOI) of 0.01. Culture supernatants were collected at the indicated time points and virus titers determined by 50% tissue culture infectious dose (TCID_50_).

### Complement component C3 ELISA

Complement C3 level was quantitated in BAL and serum by sandwich ELISA (Immunology Consultants Laboratory, Inc, USA) following manufacturer’s protocols. Total protein concentrations in BAL were determined by BCA assay (Pierce, Rockford, IL).

### Histopathology analysis

Lung tissues were fixed in 10% neutralized phosphate buffered formalin. Fixed tissues were dehydrated, embedded in paraffin, cut into 5 μm thick sections, and stained with standard hematoxylin and eosin. Histopathological lesions were scored (0 [none]-3 [extreme]) on the basis neutrophils, lymphocytes and macrophages cell infiltration and hemorrhage.

### TUNEL assay

Lung tissues were fixed in 10% neutralized phosphate buffered formalin, were embedded into paraffin, and sectioned at 5 μm following standard histology procedures. Lung sections were deparaffinized and rehydrated. The slides were incubated in 20 μg/ml proteinase K for 15 min at room temperature, and washed twice with PBS for 5 min. Terminal deoxynucleotidyl transferase dUTP nick end labeling (TUNEL) staining was performed using the in situ Apoptosis Detection Kit (KeyGen Biotech, Nanjing, China) as suggested by the manufacture. The ratio of TUNEL positive cells to the total number of lung cells was determined using a fluorescent microscope (Olympus IX51).

### Statistical analyses

Statistically significant differences between experimental groups were evaluated using the Student’s *t* test and analysis of variance (ANOVA) with the GraphPad Prism 6 software (GraphPad Software Inc., La Jolla, CA, USA). P<0.05 was considered statistically significant.

## SUPPLEMENTARY MATERIALS FIGURES AND TABLES








