# Supplementary material for: The innate immunity of guinea pigs against highly pathogenic avian influenza virus infection
Source: Oncotarget. 2017 Mar 23;8(18):30422–37. doi: 10.18632/oncotarget.16503 (PMC5444753; doi:10.18632/oncotarget.16503)
Supplement: Supplementary file 1 [file oncotarget-08-30422-s001.pdf]

The innate immunity of guinea pigs against highly pathogenic avian influenza virus infection

SUPPLEMENTARY FIGURES AND TABLES

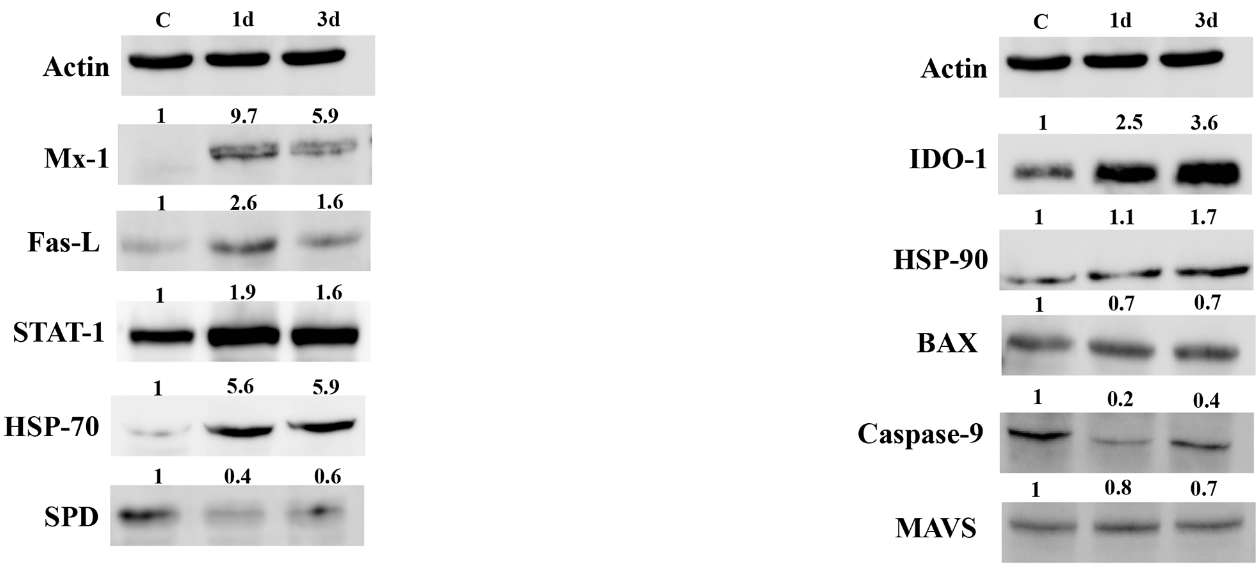

Supplementary Figure 1: Western blot analysis of selected proteins identified through iTRAQ based proteomic analysis of H5N1-infected guinea pig lungs. Levels of the indicated proteins in lung homogenates from uninfected guinea pigs (C, control), and H5N1 infected guinea pigs on day 1 and 3 post-infection were assessed by Western blot. Protein level quantification was normalized to β-actin levels as an internal control. Twenty μg total protein was loaded in each lane.

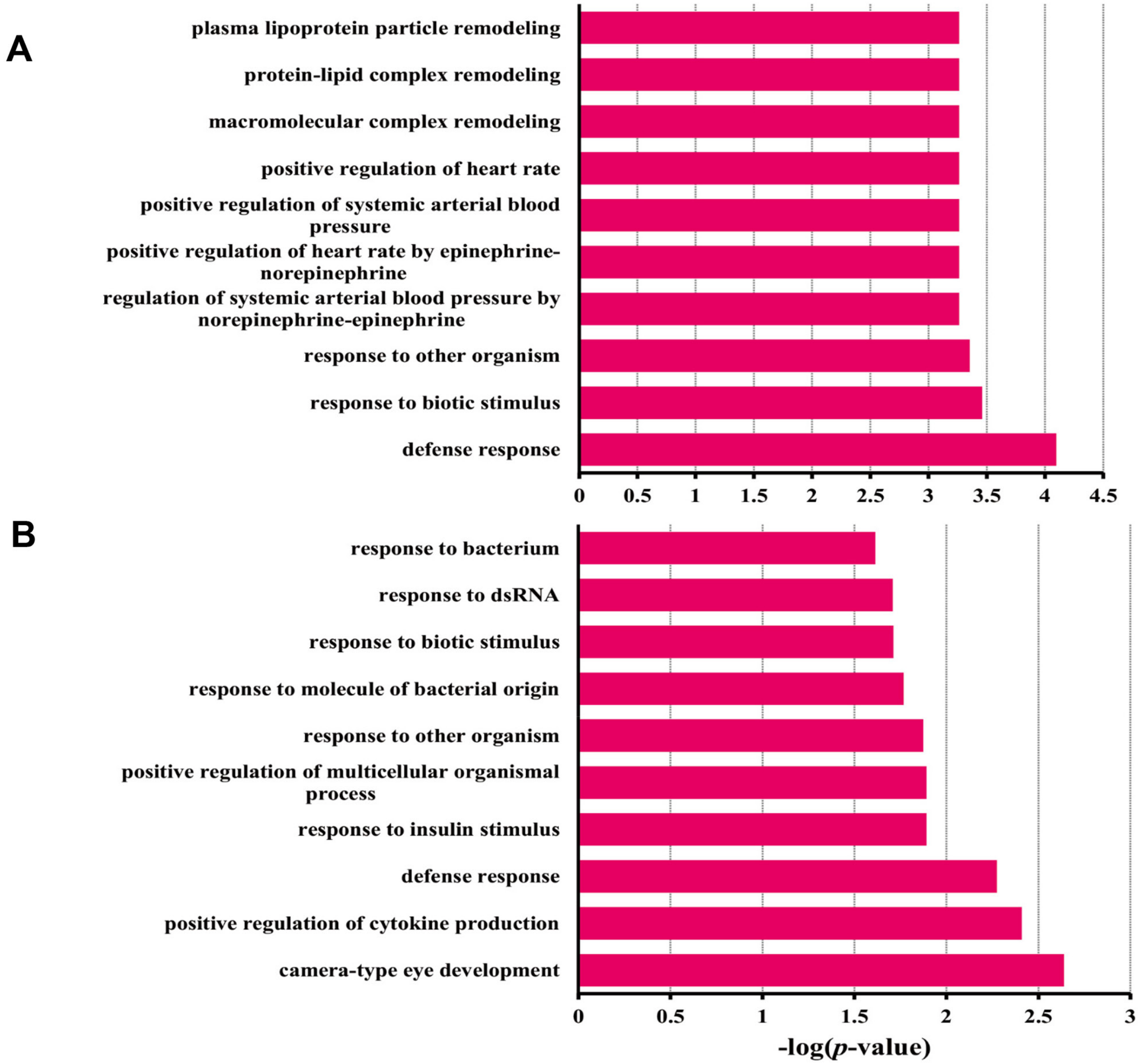

Supplementary Figure 2: Gene ontology term enrichment of differentially expressed proteins identified in the lungs of H5N1-infected guinea pigs at 1 dpi (A) and 3 dpi (B). P values were calculated using the hypergeometric test. Only the top ten GO terms are shown. For other enriched GO terms, please see Supplementary Supplementary Table 2.

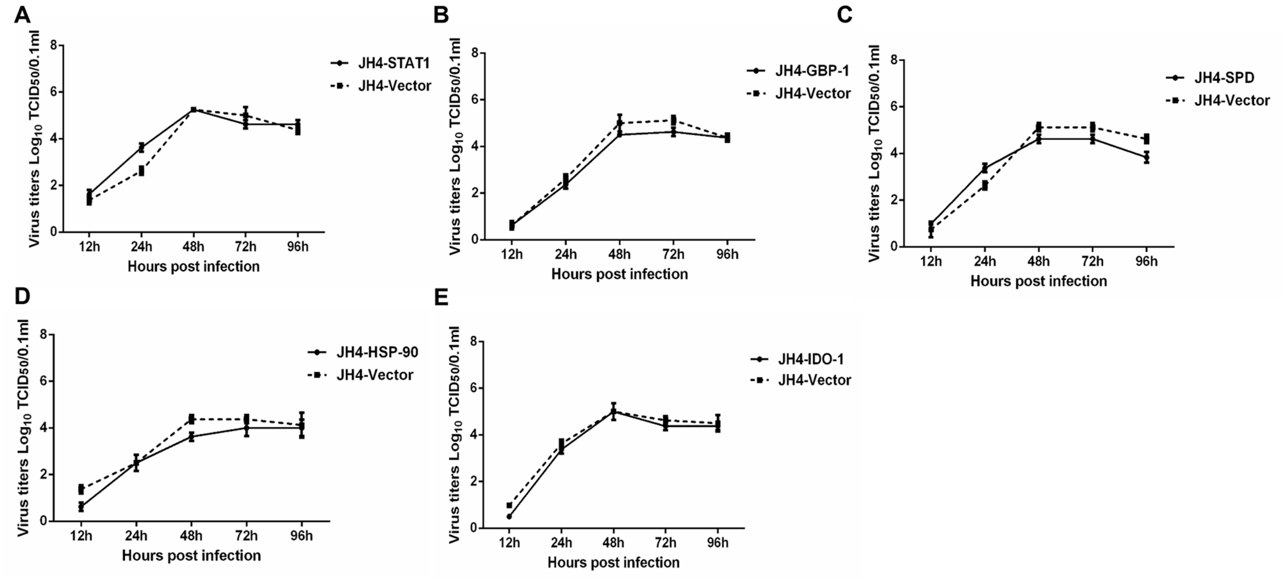

Supplementary Figure 3: Functional analysis of STAT1, GBP-1, SPD, HSP90 and IDO-1 in H5N1 infected guinea pig cells. JH4 cells were transfected with an expression vector to overexpress STAT1 **(A)**, GBP-1 **(B)**, SPD **(C)**, HSP90 **(D)** and IDO 1 **(E)**. Overexpression of vector control served as a control. Forty eight hours after transfection, cells were infected with H5N1 influenza virus at an MOI of 0.01. Culture supernatants were collected at the indicated time points to determine viral titers by $TCID_{50}$. The data represent the means of three independent experiments. Error bars represent standard deviation.

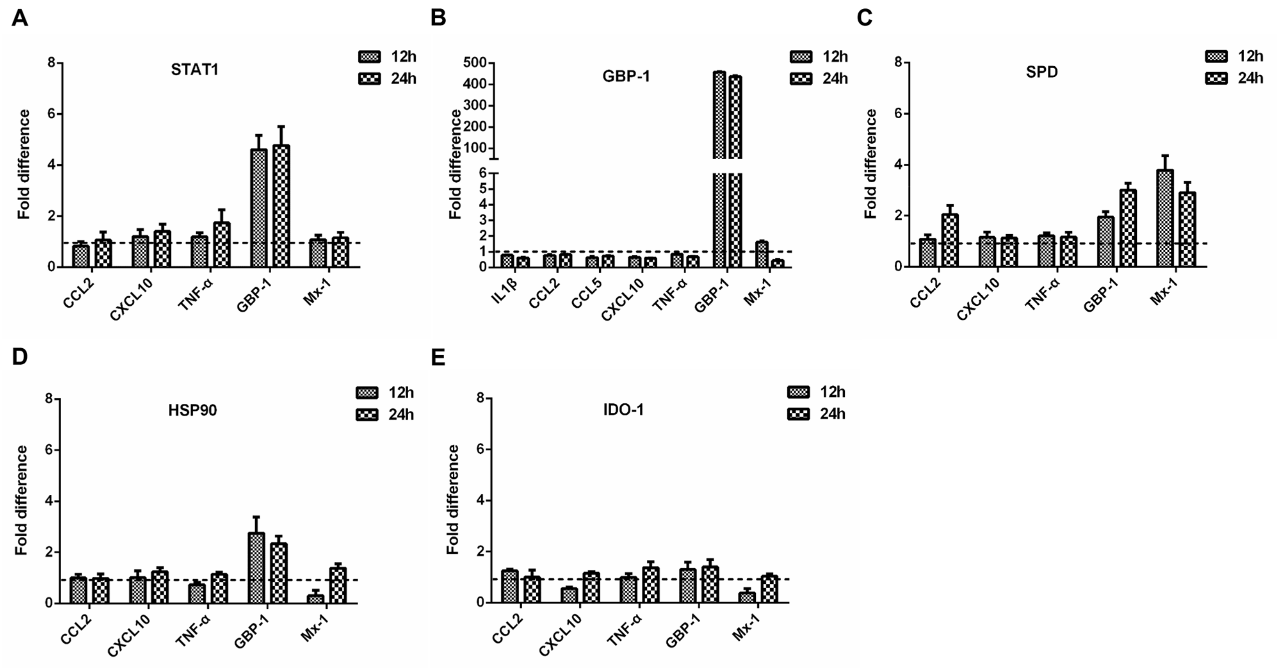

Supplementary Figure 4: Quantitative RNA analysis of STAT1, GBP-1, SPD, HSP90 and IDO-1 in H5N1 infected guinea pig cells. Figure JH4 cells were transfected with an expression vector to overexpress STAT1 **(A)**, GBP-1 **(B)**, SPD **(C)**, HSP90 **(D)** or IDO-1 **(E)**. Transfection of an empty vector served as a control. Forty-eight hours after transfection, cells were infected with H5N1 influenza virus at an MOI of 0.01. Cells were harvested at the indicated time points and cellular RNA extracted to analyze a select panel of immune related genes by quantitative RT-PCR. RNA levels were normalized to β-actin. Fold differences were determined using the$2^{-\triangle\triangle Ct}$ method as compared to cells transfected with the empty vector control. The data represent the means of three independent experiments. Error bars represent standard deviation.

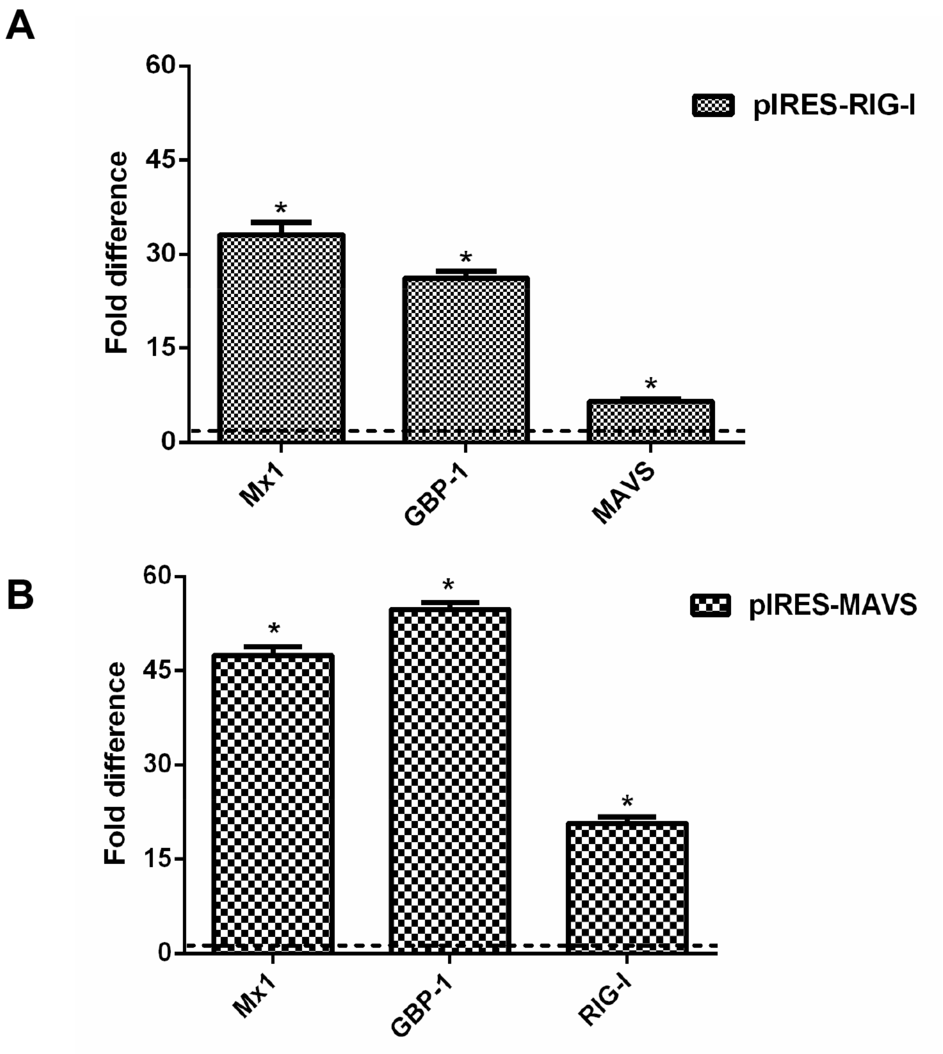

Supplementary Figure 5: Overexpression of RIG-I and MAVS in guinea pig cells. JH4 cells were transfected with an expression vector to overexpress RIG-I **(A)** and MAVS **(B)**. Transfection with an empty vector served as a control. Forty eight hours after transfection, cells were harvested. RNA harvested from cells was used to evaluate expression of the indicated genes by quantitative RT-PCR. RNA levels were normalized to β-actin. The fold difference from the qRT-PCR was determined using the $2^{-\triangle\triangle Ct}$ method and are expressed as fold induction as compared to the vector control. The data represent the means of three independent experiments. Error bars represent standard deviation.

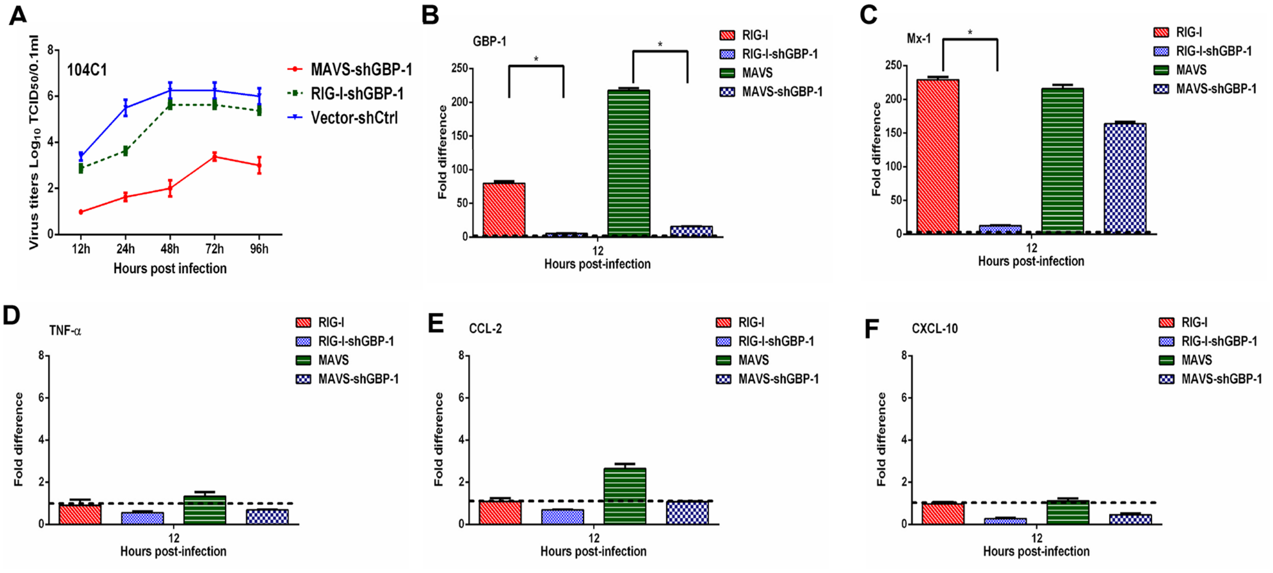

Supplementary Figure 6: GBP-1 is required for RIG-I mediated inhibition of H5N1 replication in guinea pig 104C1 cells. 104C1 cells were transfected with an expression plasmid to overexpress RIG-I, MAVS, or vector control and either a shRNA targeting GBP-1 (shGBP-1) or a shRNA control (shCtrl). Forty eight hours after transfection, cells were infected with H5N1 virus at an MOI of 0.01. Culture supernatants were collected at the indicated time points post infection to determine virus titer by $TCID_{50}$ **(panel A)**. RNA harvested from cells was used to evaluate expression of the indicated genes by quantitative RT-PCR **(panels B-F)**. RNA levels were normalized to β-actin. The fold difference from the qRT-PCR was determined using the $2^{-\triangle\triangle Ct}$ method and are expressed as fold induction as compared to cells overexpressing RIG-I or MAVS co-transfected with the shRNA control. The data represent the means of three independent experiments. Error bars represent standard deviation.

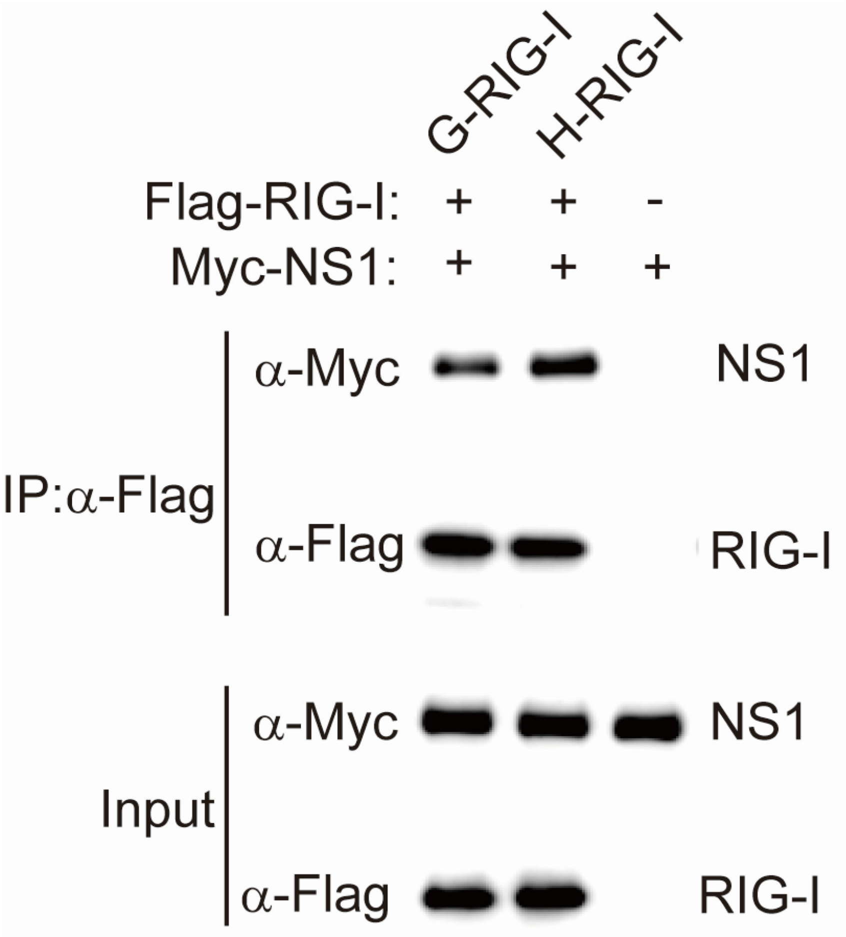

Supplementary Figure 7: Guinea pig RIG-I interacts with NS1 protein. NS1 was co-transfected with guinea pig RIG-I or human RIG-I in 293T cells. Cell lysates were immunoprecipitated with anti-Flag antibody followed by immunoblotting with anti-Myc and anti-Flag antibodies as indicated. Input, 2% whole-cell lysate used for IP.

Supplementary Table 1: Statistically significant differentially expressed proteins identified by iTRAQ analysis of lungs infected with H5N1 AIV.

See Supplementary File 1

Supplementary Table 2: Significantly enriched GO terms among differentially expressed proteins.

See Supplementary File 2

Supplementary Table 3: Primer sequences used for real time RT-PCR analyses.

See Supplementary File 3
