# Supplementary material for: The innate immunity of guinea pigs against highly pathogenic avian influenza virus infection
Source: Oncotarget. 2017 Mar 23;8(18):30422–37. doi: 10.18632/oncotarget.16503 (PMC5444753; doi:10.18632/oncotarget.16503)
Supplement: Supplementary file 2 [file oncotarget-08-30422-s002.docx]

**Table S1.** **Statistically significant differentially expressed proteins identified by iTRAQ analysis of lungs infected with H5N1 AIV**

| **Accession** | **Protein name** | **Ratio** | | **Cov** | **Function** |
| --- | --- | --- | --- | --- | --- |
|  |  | 1d | 3d |  |  |
| Cluster 1: Up-regulated at day 1 and/or day 3 post-infection (84) | |  |  |  |  |
|  | |  |  |  |  |
| gi\|348554277 | PREDICTED: transmembrane emp24 domain-containing protein 2-like | **1.774** | 0.882 | 8 | anatomical structure development |
| gi\|348556421 | PREDICTED: proteasome assembly chaperone 1-like isoform 1 | **1.594** | 0.844 | 5.5 | anatomical structure formation |
| gi\|348572974 | ***PREDICTED: annexin A1-like*** | **1.529** | 0.989 | 39.6 | Defense  response |
| gi\|348558631 | PREDICTED: dihydropteridine reductase-like | **1.513** | 0.957 | 9.7 | response to stimulus |
| gi\|348571341 | PREDICTED: proteasome subunit beta type-2-like isoform 1 | **1.501** | 1.342 | 12.9 | response to stimulus |
| gi\|348557622 | PREDICTED: apolipoprotein E-like | **1.506** | 1.194 | 18.1 | response to external stimulus |
| gi\|348577433 | PREDICTED: proteasome activator complex subunit 2-like | **1.599** | 1.152 | 15.5 | response to stimulus |
| gi\|348578808 | PREDICTED: NADH dehydrogenase [ubiquinone] 1 alpha subcomplex subunit 4-like | **1.64** | 1.173 | 14.6 | oxidation reduction |
| gi\|348556648 | PREDICTED: actin-related protein 2/3 complex subunit 4-like | **1.662** | 1.307 | 29.2 | cellular component organization |
| gi\|348586904 | PREDICTED: transmembrane emp24 domain-containing protein 5-like | **1.621** | 0.918 | 5.3 | localization |
| gi\|290543394 | eosinophil granule major basic protein 1 preproprotein | **2.098** | 1.08 | 8.6 | _ |
| gi\|348575640 | PREDICTED: tumor necrosis factor receptor superfamily member 6-like | **2.178** | 1.368 | 3 | response to external stimulus |
| gi\|348554649 | PREDICTED: tryptophanyl-tRNA synthetase, cytoplasmic | **1.675** | 1.785 | 30.3 | anatomical structure development |
| gi\|348573312 | PREDICTED: synaptojanin-2-binding protein-like | **1.531** | 1.36 | 9.7 | biological regulation |
| gi\|348575794 | PREDICTED: chloride intracellular channel protein 5-like | **1.686** | 1.076 | 21.6 | anatomical structure development |
| gi\|348563575 | PREDICTED: vitamin D-binding protein-like | **2.117** | 1.017 | 17.1 | response to external stimulus |
| gi\|348551037 | PREDICTED: ribonuclease inhibitor-like | **1.523** | 1.166 | 6.4 | biological regulation |
| gi\|348582214 | PREDICTED: fibrinogen alpha chain-like | **3.556** | 1.27 | 12.5 | response to stimulus |
| *gi\|348550493 | PREDICTED: HLA class I histocompatibility antigen, B-40 alpha chain-like | **1.887** | **1.519** | 2.7 | response to stimulus |
| gi\|348566569 | PREDICTED: prenylcysteine oxidase-like | **1.58** | 1.167 | 7.9 | localization |
| gi\|348561075 | PREDICTED: mitochondrial 2-oxoglutarate/malate carrier protein-like isoform 1 | **1.632** | 1.531 | 15.9 | localization |
| gi\|348586868 | PREDICTED: interferon-induced guanylate-binding protein 1-like | **3.811** | 1.616 | 8.5 | biological regulation |
| *gi\|348586135 | PREDICTED: LOW QUALITY PROTEIN: signal transducer and activator of transcription 1-like | **2.815** | **1.66** | 20.2 | response to biotic stimulus |
| gi\|348569855 | PREDICTED: long-chain fatty acid transport protein 4-like | **1.9** | 2.185 | 7.8 | response to external stimulus |
| gi\|348555647 | PREDICTED: 40S ribosomal protein S27-like | **1.73** | 1.566 | 9.5 | response to stimulus |
| gi\|348554659 | PREDICTED: heat shock protein HSP 90-alpha-like | **1.648** | 1.335 | 31.7 | response to external stimulus |
| gi\|348584148 | PREDICTED: 40S ribosomal protein S15a-like isoform 1 | **1.563** | 0.802 | 20 | response to biotic stimulus |
| gi\|348576344 | PREDICTED: 60S ribosomal protein L10a-like | **1.517** | 1.463 | 3.7 | anatomical structure development |
| gi\|348552043 | PREDICTED: cAMP-dependent protein kinase catalytic subunit alpha-like isoform 1 | **1.593** | 1.032 | 7.1 | response to stimulus |
| gi\|348567833 | PREDICTED: tax1-binding protein 3-like | **1.622** | 1.067 | 6.5 | anatomical structure development |
| *gi\|348557750 | ***PREDICTED: indoleamine 2,3-dioxygenase 1-like*** | **2.921** | **1.648** | 17.8 | defense response |
| gi\|348556984 | PREDICTED: bone marrow stromal antigen 2-like | **5.286** | 2.838 | 11.8 | _ |
| gi\|348563757 | PREDICTED: 5-hydroxytryptamine receptor 2C | **1.576** | 2.847 | 1.5 | locomotion |
| gi\|348580753 | ***PREDICTED: lysozyme C-like*** | **1.544** | 0.999 | 22.3 | defense response |
| gi\|348578633 | PREDICTED: protein FAM3C-like | **1.616** | 1.269 | 7 | multicellular organismal process |
| gi\|348580982 | PREDICTED: CD63 antigen-like | **1.516** | 1.189 | 5.9 | locomotion |
| gi\|348554473 | ***PREDICTED: alpha-1-antichymotrypsin-like*** | **1.831** | 0.931 | 9.1 | defense response |
| gi\|348562897 | PREDICTED: glucose-6-phosphate isomerase-like | **1.876** | 1.127 | 7.3 | response to stimulus |
| *gi\|348556357 | ***PREDICTED: interferon-induced GTP-binding protein Mx2-like, partial*** | **4.774** | **3.674** | 11.2 | defense response |
| gi\|348550587 | PREDICTED: leukocyte elastase inhibitor-like | **2.377** | 0.977 | 13.8 | regulation of hydrolase activity |
| gi\|348580125 | PREDICTED: methyltransferase-like protein 7A-like isoform 1 | **1.583** | 1.333 | 15.1 | one-carbon metabolic process |
| gi\|290543340 | ***complement C3 preproprotein*** | **1.86** | 0.983 | 15.6 | defense response |
| gi\|348570851 | PREDICTED: F-actin-capping protein subunit beta-like | **1.637** | 1.452 | 16.8 | negative regulation of cytoskeleton organization |
| gi\|348583435 | PREDICTED: dolichyl-phosphate beta-glucosyltransferase-like isoform 1 | **1.884** | 0.802 | 8.6 | developmental process |
| gi\|348556672 | PREDICTED: LIM and cysteine-rich domains protein 1 | **1.555** | 1.363 | 4 | biological regulation |
| gi\|348552098 | PREDICTED: ATP-dependent RNA helicase DDX39A | **1.895** | 1.344 | 14.1 | localization |
| gi\|348582342 | ***PREDICTED: lactotransferrin-like*** | **5.104** | 0.887 | 24.7 | defense response |
| gi\|348586419 | ***PREDICTED:*** ***protein S100-A8-like*** | **2.956** | 2.058 | 12.4 | defense response |
| gi\|348565037 | PREDICTED: ras-related protein Rab-1B-like | **1.796** | 1.077 | 39.3 | localization |
| *gi\|348571756 | PREDICTED: LIM and senescent cell antigen-like-containing domain protein 1-like isoform 1 | **1.886** | **1.812** | 2.8 | developmental process |
| gi\|348578563 | PREDICTED: serum paraoxonase/lactonase 3-like | **1.594** | 1.157 | 6.1 | response to stimulus |
| gi\|290542323 | fatty acid-binding protein, epidermal | **1.506** | 1.329 | 23.7 | anatomical structure development |
| gi\|348559961 | PREDICTED: 116 kDa U5 small nuclear ribonucleoprotein component-like | **1.653** | 0.994 | 7.8 | RNA splicing |
| gi\|348563112 | PREDICTED: alpha-1B-glycoprotein-like | **3.045** | 0.814 | 8.1 | _ |
| gi\|348562125 | ***PREDICTED: eosinophil peroxidase-like*** | **1.73** | 1.467 | 8.3 | defense response |
| gi\|348558126 | PREDICTED: carbonyl reductase [NADPH] 2-like | **1.738** | 1.289 | 8.2 | oxidation reduction |
| gi\|348572031 | PREDICTED: glucosamine 6-phosphate N-acetyltransferase-like | **2.687** | 1.384 | 6 | anatomical structure development |
| gi\|290542307 | eosinophil granule major basic protein 2 preproprotein | **1.512** | 1.146 | 8.5 | response to stimulus |
| gi\|348552740 | PREDICTED: rab GDP dissociation inhibitor alpha-like isoform 1 | **1.637** | 1.487 | 22.1 | response to stimulus |
| gi\|348568716\| | PREDICTED: proteasome subunit alpha type-2-like | **1.585** | 1.19 | 29.1 | response to biotic stimulus |
| gi\|348575580 | PREDICTED: inter-alpha-trypsin inhibitor heavy chain H2-like | **1.672** | 1.459 | 1.6 | regulation of hydrolase activity |
| gi\|348572792 | ***PREDICTED: haptoglobin-like*** | **2.613** | 1.404 | 6.7 | defense response |
| gi\|348562522 | ***PREDICTED: myeloperoxidase*** | **3.164** | 0.949 | 11.7 | defense response |
| gi\|348581656 | PREDICTED: ceruloplasmin-like | **2.168** | 1.039 | 5.5 | response to stimulus |
| gi\|348567657 | PREDICTED: vitronectin-like | 0.844 | **2.065** | 3.1 | smooth muscle cell migration |
| gi\|348554740 | PREDICTED: BAG family molecular chaperone regulator 5-like | 1.063 | **1.618** | 5.1 | _ |
| gi\|348585583 | PREDICTED: ubiquitin-conjugating enzyme E2 E3-like | 1.028 | **4.825** | 5.7 | chromatin organization |
| gi\|348580966 | PREDICTED: cyclin-dependent kinase 2 isoform 1 | 1.387 | **1.634** | 9.4 | response to insulin stimulus |
| gi\|348560943 | PREDICTED: 60S ribosomal protein L26-like | 1.103 | **1.711** | 15.9 | ribosome biogenesis |
| gi\|348552884 | PREDICTED: carbonyl reductase [NADPH] 1-like | 0.952 | **1.55** | 10.5 | cofactor metabolic process |
| gi\|348566427 | PREDICTED: S-adenosylmethionine synthase isoform type-2 | 1.462 | **1.546** | 10.4 | response to organic substance |
| gi\|5835998 | NADH dehydrogenase subunit 4 | 1.42 | **2.376** | 1.5 | respiratory electron transport chain |
| gi\|348588935 | PREDICTED: ras-related protein Rab-5A-like | 0.996 | **1.55** | 30.7 | smooth muscle cell migration |
| gi\|348557124 | PREDICTED: 182 kDa tankyrase-1-binding protein-like | 0.821 | **1.559** | 3.5 | cellular process |
| gi\|348552770 | PREDICTED: isocitrate dehydrogenase [NAD] subunit gamma, mitochondrial-like | 1.319 | **1.578** | 2 | cellular respiration |
| gi\|348553917 | PREDICTED: aldo-keto reductase family 1 member C1 homolog | 1.605 | **1.942** | 5.6 | response to chemical stimulus |
| gi\|348582524 | PREDICTED: fibrinogen beta chain-like isoform 1 | 4.27 | **1.617** | 16.2 | response to chemical stimulus |
| gi\|348569044 | PREDICTED: superkiller viralicidic activity 2-like | -- | **11.064** | 1.9 | cellular process |
| gi\|348572978 | PREDICTED: 40S ribosomal protein S6-like | 1.269 | **1.51** | 17.3 | interphase of mitotic cell cycle |
| gi\|348574784 | PREDICTED: caspase recruitment domain-containing protein 9-like | -- | **10.406** | 6.3 | positive regulation of cytokine production |
| gi\|348577524 | PREDICTED: myosin-6-like | 0.814 | **1.674** | 6.3 | cellular component organization |
| gi\|290543336 | alpha-crystallin B chain | 1.006 | **1.682** | 12.6 | camera-type eye development |
| gi\|348583329 | PREDICTED: sorting nexin-2-like | 1.336 | **1.783** | 2.7 | cellular process |
| gi\|348581374 | PREDICTED: phosphatidate cytidylyltransferase 2-like | 1.987 | **2.413** | 5.2 | organophosphate metabolic process |
|  |  |  |  |  |  |
| Cluster 2: Down-regulated at day 1 and/or day 3 post-infection (156) | |  |  |  |  |
|  | |  |  |  |  |
| gi\|348550543 | PREDICTED: small glutamine-rich tetratricopeptide repeat-containing protein alpha-like | **0.561** | 1.065 | 4.1 | _ |
| gi\|348555199 | PREDICTED: hypothetical protein LOC100722952 | **0.473** | 0.812 | 6.7 | response to stimulus |
| gi\|348562229 | PREDICTED: collagen alpha-1(I) chain isoform 1 | **0.589** | 1.044 | 4.6 | response to external stimulus |
| gi\|348575684 | PREDICTED: transcription factor A, mitochondrial-like | **0.647** | 0.977 | 13.7 | biological regulation |
| gi\|348570354 | PREDICTED: UV excision repair protein RAD23 homolog B-like | **0.66** | 1.117 | 11.7 | response to stimulus |
| gi\|348555631 | PREDICTED: cytochrome c oxidase subunit 5A, mitochondrial-like | **0.438** | 0.696 | 34 | oxidation reduction |
| gi\|348554581 | PREDICTED: chromobox protein homolog 3-like | **0.647** | 0.885 | 43.2 | biological regulation |
| gi\|348550658 | PREDICTED: calcyphosin-like isoform 1 | **0.522** | 0.76 | 30.2 | biological regulation |
| gi\|348585481 | PREDICTED: hematological and neurological expressed 1-like protein-like | **0.443** | 1.072 | 7.4 | _ |
| gi\|348557600 | PREDICTED: zinc finger protein 428-like isoform 1 | **0.526** | 0.757 | 5.7 | _ |
| gi\|348581480 | PREDICTED: 5~-3~ exoribonuclease 2-like | **0.549** | 1.042 | 3.3 | male gamete generation |
| gi\|348558172 | PREDICTED: hematological and neurological expressed 1 protein-like | **0.49** | 1.018 | 6.9 | _ |
| gi\|348554251 | PREDICTED: thymosin beta-4-like | **0.292** | 0.785 | 15.9 | locomotion |
| gi\|348582462 | PREDICTED: coiled-coil domain-containing protein 50-like | **0.647** | 0.975 | 3.9 | _ |
| gi\|348563773 | PREDICTED: myosin regulatory light polypeptide 9-like | **0.36** | 0.618 | 33.7 | response to external stimulus |
| *gi\|348581384 | PREDICTED: mitochondrial antiviral-signaling protein-like | **0.358** | **0.66** | 2.1 | response to biotic stimulus |
| gi\|348576388 | PREDICTED: prefoldin subunit 6-like | **0.365** | 0.78 | 19.1 | anatomical structure formation |
| *gi\|348574574 | PREDICTED: protein dpy-30 homolog | **0.368** | **0.564** | 29.3 | localization |
| gi\|348570542 | PREDICTED: LOW QUALITY PROTEIN: sorting nexin-12-like | **0.648** | 1.012 | 13.6 | localization |
| gi\|348582382 | PREDICTED: microtubule-associated protein 4-like | **0.593** | 1.192 | 31.9 | _ |
| *gi\|348562789 | PREDICTED: LOW QUALITY PROTEIN: protein phosphatase 1 regulatory subunit 14A-like | **0.43** | **0.663** | 20.1 | metabolic process |
| gi\|348567266 | PREDICTED: SPARC-like protein 1-like | **0.449** | 0.743 | 4.6 | _ |
| gi\|348581075 | ***PREDICTED: poly(rC)-binding protein 2 isoform 1*** | **0.649** | 1.122 | 24.8 | defense response |
| gi\|348563857 | PREDICTED: charged multivesicular body protein 4b-like | **0.626** | 0.874 | 19.6 | localization |
| gi\|348570240 | PREDICTED: tropomyosin beta chain-like isoform 3 | **0.332** | 0.555 | 41.5 | regulation of systemic arterial blood pressure by norepinephrine-epinephrine |
| gi\|348557392 | PREDICTED: NADH dehydrogenase [ubiquinone] flavoprotein 2, mitochondrial-like | **0.647** | 0.753 | 13.3 | anatomical structure development |
| gi\|348583168 | PREDICTED: response gene to complement 32 protein-like | **0.366** | 0.851 | 6.9 | biological regulation |
| gi\|348566079 | PREDICTED: histone H1.1-like | **0.658** | 0.413 | 5.6 | male gamete generation |
| gi\|348570464 | PREDICTED: SH3 domain-binding glutamic acid-rich-like protein-like | **0.53** | 0.856 | 36.2 | _ |
| gi\|348571158 | PREDICTED: stathmin-like | **0.486** | 1.122 | 22.8 | response to biotic stimulus |
| gi\|348578919 | PREDICTED: V-type proton ATPase subunit F-like | **0.666** | 0.718 | 52.1 | anatomical structure development |
| gi\|348578229 | PREDICTED: leiomodin-1-like | **0.56** | 0.662 | 2.7 | multicellular organismal process |
| gi\|348555583 | PREDICTED: tropomyosin alpha-4 chain-like isoform 4 | **0.385** | 1.069 | 31.5 | regulation of systemic arterial blood pressure by norepinephrine-epinephrine |
| gi\|348554365 | PREDICTED: endoplasmic reticulum resident protein 29-like | **0.657** | 0.913 | 33.7 | response to stimulus |
| gi\|348565713 | PREDICTED: protein CWC15 homolog | **0.459** | 0.729 | 4.8 | regulation of innate immune response |
| gi\|348577411 | PREDICTED: NEDD8-like | **0.501** | 0.762 | 13.6 | response to stimulus |
| gi\|348581087 | PREDICTED: prefoldin subunit 5-like | **0.527** | 0.981 | 21.4 | anatomical structure development |
| gi\|348555621 | PREDICTED: reticulocalbin-2-like | **0.532** | 0.945 | 10.7 | _ |
| gi\|348575706 | PREDICTED: heterogeneous nuclear ribonucleoprotein H3-like | **0.507** | 0.691 | 3.8 | RNA splicing |
| gi\|348581271 | PREDICTED: NSFL1 cofactor p47-like isoform 1 | **0.555** | 0.829 | 30.8 | cellular component organization |
| gi\|348575478 | PREDICTED: leucine-rich repeat flightless-interacting protein 2 isoform 1 | **0.543** | 0.663 | 5.8 | biological regulation |
| *gi\|348564597 | ***PREDICTED: aminoacyl tRNA synthase complex-interacting multifunctional protein 1-like*** | **0.595** | **0.642** | 13.7 | defense response |
| gi\|348572578 | PREDICTED: tubulin polymerization-promoting protein family member 3-like | **0.44** | 0.679 | 19.9 | cellular component organization |
| gi\|348570356 | PREDICTED: collagen alpha-1(V) chain-like | **0.501** | 1.181 | 1.5 | response to external stimulus |
| gi\|348575003 | PREDICTED: clathrin light chain B-like | **0.404** | 0.816 | 14.8 | multicellular organismal process |
| gi\|348570272 | PREDICTED: clathrin light chain A-like isoform 3 | **0.534** | 0.857 | 25 | response to external stimulus |
| gi\|348565947 | PREDICTED: uncharacterized protein KIAA1462-like | **0.566** | 0.919 | 3.3 | _ |
| gi\|348561916 | PREDICTED: brain acid soluble protein 1-like | **0.379** | 0.81 | 33.6 | _ |
| gi\|348572074 | PREDICTED: peptidyl-prolyl cis-trans isomerase FKBP3-like | **0.564** | 0.864 | 18.3 | peptidyl-amino acid modification |
| gi\|348567967 | PREDICTED: 60S ribosomal protein L23a-like isoform 1 | **0.568** | 0.71 | 14.1 | anatomical structure development |
| gi\|348554235 | PREDICTED: rab11 family-interacting protein 1-like | **0.496** | 0.936 | 4.4 | localization |
| gi\|348562877 | PREDICTED: ATP synthase-coupling factor 6, mitochondrial-like | **0.587** | 0.931 | 17.6 | regulation of transport |
| gi\|348565025 | PREDICTED: barrier-to-autointegration factor-like | **0.531** | 0.806 | 29.2 | response to biotic stimulus |
| gi\|348556369 | PREDICTED: cystatin-B-like | **0.614** | 0.753 | 19.4 | response to stimulus |
| gi\|348555581 | PREDICTED: tropomyosin alpha-4 chain-like isoform 3 | **0.449** | 0.918 | 41.1 | regulation of systemic arterial blood pressure by norepinephrine-epinephrine |
| *gi\|348564073 | PREDICTED: WAP four-disulfide core domain protein 2-like | **0.312** | **0.628** | 4.9 | regulation of hydrolase activity |
| gi\|348556419 | PREDICTED: non-histone chromosomal protein HMG-14-like | **0.368** | 0.686 | 22.8 | response to stimulus |
| gi\|348570758 | PREDICTED: protein FAM54B-like | **0.504** | 0.737 | 3.7 | _ |
| *gi\|348574155 | PREDICTED: transgelin-like | **0.633** | **0.491** | 28.9 | anatomical structure development |
| gi\|348575940 | PREDICTED: U1 small nuclear ribonucleoprotein C-like | **0.558** | 0.836 | 6.1 | RNA splicing |
| gi\|348574167 | PREDICTED: LOW QUALITY PROTEIN: cell adhesion molecule 1-like | **0.561** | 0.734 | 2.8 | anatomical structure development |
| gi\|348560678 | ***PREDICTED: pulmonary surfactant-associated protein D-like*** | **0.494** | 0.897 | 12.5 | defense response |
| gi\|348559512 | PREDICTED: reticulocalbin-3-like | **0.53** | 0.901 | 23 | _ |
| gi\|348569468 | PREDICTED: galectin-1-like | **0.622** | 0.715 | 30.4 | response to external stimulus |
| gi\|348554706 | PREDICTED: cysteine-rich protein 2-like | **0.435** | 0.815 | 18.3 | anatomical structure development |
| gi\|348585327 | PREDICTED: 39S ribosomal protein L40, mitochondrial-like | **0.58** | 0.534 | 14.6 | developmental process |
| gi\|348553881 | PREDICTED: hippocalcin-like protein 1-like | **0.54** | 0.845 | 13 | _ |
| gi\|348559939 | PREDICTED: nucleobindin-2-like | **0.647** | 0.682 | 6.2 | biological regulation |
| gi\|348553439 | PREDICTED: cytochrome b-c1 complex subunit 6, mitochondrial-like | **0.313** | 0.804 | 28.3 | oxidation reduction |
| gi\|348557434 | PREDICTED: SPARC-like | **0.449** | 1.075 | 5.3 | response to biotic stimulus |
| gi\|348578989 | PREDICTED: actin-binding LIM protein 1 isoform 1 | **0.626** | 0.776 | 6.2 | response to external stimulus |
| gi\|348588893 | PREDICTED: neurocalcin-delta-like | **0.54** | 0.845 | 17.1 | localization |
| gi\|348577387 | PREDICTED: prothymosin alpha-like | **0.327** | 0.734 | 14.3 | _ |
| gi\|348562283 | PREDICTED: chromobox protein homolog 1-like | **0.471** | 0.672 | 17.3 | biological regulation |
| gi\|348583882 | PREDICTED: lysosome membrane protein 2-like | **0.622** | 0.813 | 10 | cellular process |
| gi\|348578129 | PREDICTED: nuclear ubiquitous casein and cyclin-dependent kinases substrate-like | **0.315** | 0.773 | 17.7 | _ |
| gi\|348568450 | PREDICTED: eukaryotic translation initiation factor 4H-like isoform 1 | **0.553** | 0.983 | 13.8 | multi-organism process |
| gi\|348558286 | PREDICTED: THO complex subunit 4-like | **0.548** | 0.89 | 10.7 | localization |
| gi\|348573123 | PREDICTED: enhancer of rudimentary homolog | **0.625** | 0.934 | 10.6 | nucleoside metabolic process |
| gi\|348581257 | PREDICTED: peptidyl-prolyl cis-trans isomerase FKBP1A-like | **0.53** | 0.852 | 36.1 | cardiac muscle tissue morphogenesis |
| gi\|348587090 | PREDICTED: pre-mRNA-splicing factor SPF27-like | **0.548** | 0.958 | 11.1 | RNA splicing |
| gi\|348551765 | PREDICTED: heterogeneous nuclear ribonucleoprotein A/B-like | **0.626** | 0.782 | 21.4 | anatomical structure development |
| gi\|348573171 | PREDICTED: hypothetical protein LOC100724485 | **0.329** | 0.858 | 10.3 | _ |
| gi\|348578547 | PREDICTED: collagen alpha-2(I) chain-like | **0.308** | 0.796 | 6.5 | response to external stimulus |
| gi\|348585449 | PREDICTED: serine/arginine-rich splicing factor 9-like | **0.445** | 0.769 | 13.6 | localization |
| gi\|348584162 | PREDICTED: nuclear distribution protein nudE homolog 1-like | **0.621** | 0.769 | 6.9 | locomotion |
| gi\|348564593 | PREDICTED: nephronectin-like | **0.631** | 0.786 | 1.4 | anatomical structure development |
| gi\|348576508 | ***PREDICTED: allograft inflammatory factor 1-like*** | **0.6** | 0.832 | 17.7 | defense response |
| gi\|348558204 | PREDICTED: Na(+)/H(+) exchange regulatory cofactor NHE-RF1-like | **0.545** | 0.941 | 24.4 | locomotion |
| gi\|348585525 | PREDICTED: Na(+)/H(+) exchange regulatory cofactor NHE-RF2-like isoform 1 | **0.63** | 0.937 | 7.7 | anatomical structure development |
| gi\|348572868 | PREDICTED: osteoclast-stimulating factor 1-like | **0.564** | 0.756 | 23 | multicellular organismal process |
| gi\|348588679 | PREDICTED: tumor protein D52-like isoform 1 | **0.558** | 0.814 | 6.7 | anatomical structure development |
| gi\|348550099 | PREDICTED: PDZ domain-containing protein GIPC3-like | **0.53** | 0.737 | 2.9 | _ |
| gi\|348557452 | PREDICTED: reticulocalbin-1-like | **0.535** | 1.019 | 22.9 | anatomical structure development |
| gi\|348588663 | PREDICTED: acidic leucine-rich nuclear phosphoprotein 32 family member A-like | **0.581** | 1.108 | 23.7 | biological regulation |
| gi\|348588407 | PREDICTED: cytochrome b-c1 complex subunit 7-like | **0.531** | 0.735 | 17.1 | oxidation reduction |
| gi\|348581908 | PREDICTED: dystroglycan-like | **0.647** | 1.041 | 2.8 | locomotion |
| *gi\|348586133 | PREDICTED: serum deprivation-response protein-like | **0.495** | **0.614** | 21.1 | biological regulation |
| *gi\|348583213 | PREDICTED: dihydropyrimidinase-related protein 3-like | **0.662** | **0.557** | 14.3 | response to external stimulus |
| gi\|348579354 | PREDICTED: hepatoma-derived growth factor-like | **0.461** | 0.809 | 26.2 | _ |
| gi\|348550680 | PREDICTED: far upstream element-binding protein 2 | **0.656** | 1.137 | 14.3 | localization |
| gi\|348579273 | PREDICTED: hypothetical protein LOC100724959 | **0.552** | 0.91 | 13.6 | multicellular organismal process |
| gi\|348585359 | PREDICTED: ran-specific GTPase-activating protein-like | **0.591** | 0.794 | 5.4 | regulation of hydrolase activity |
| gi\|348562139 | PREDICTED: serine/arginine-rich splicing factor 1-like isoform 2 | **0.635** | 0.816 | 32.8 | striated muscle contraction |
| gi\|348582596 | PREDICTED: uncharacterized protein KIAA1143 homolog | **0.431** | 1.03 | 7.1 | _ |
| *gi\|348584224 | PREDICTED: transmembrane protein 119-like | **0.199** | **0.587** | 6 | _ |
| gi\|348568564 | PREDICTED: 28 kDa heat- and acid-stable phosphoprotein-like | **0.645** | 0.976 | 16.6 | _ |
| gi\|348576084 | PREDICTED: proactivator polypeptide-like isoform 1 | **0.33** | 0.807 | 10.6 | developmental process |
| gi\|348552412 | PREDICTED: protein SET-like | **0.497** | 0.711 | 18.5 | negative regulation of biological process |
| gi\|348558666 | PREDICTED: coiled-coil domain-containing protein 124-like isoform 1 | **0.639** | 0.801 | 22.1 | _ |
| gi\|348555537 | PREDICTED: thioredoxin-like | **0.666** | 0.729 | 21 | response to stimulus |
| gi\|348567541 | PREDICTED: hypothetical protein LOC100729908 | **0.625** | 0.907 | 4.7 | protein catabolic process |
| gi\|348579734 | PREDICTED: pre-B-cell leukemia transcription factor-interacting protein 1-like | **0.542** | 0.817 | 2.2 | developmental process |
| gi\|348556189 | ***PREDICTED: CD44 antigen-like*** | **0.625** | 0.953 | 3.4 | defense response |
| gi\|348586078 | PREDICTED: translin-like | **0.619** | 0.892 | 5.7 | DNA metabolic process |
| gi\|348553780 | PREDICTED: glutaredoxin-related protein 5, mitochondrial-like | **0.636** | 1.179 | 21.2 | anatomical structure development |
| gi\|348572728 | PREDICTED: PDZ and LIM domain protein 5-like isoform 1 | **0.581** | 1.003 | 7.2 | anatomical structure development |
| gi\|348578907 | PREDICTED: calumenin-like isoform 2 | **0.506** | 0.898 | 28.3 | response to external stimulus |
| *gi\|348564450 | PREDICTED: podocalyxin-like | **0.373** | **0.617** | 1.9 | locomotion |
| gi\|348584690 | PREDICTED: ubiquitin-conjugating enzyme E2 L3-like | **0.492** | 1.79 | 29.3 | response to stimulus |
| gi\|348559352 | PREDICTED: nucleobindin-1-like | **0.505** | 0.792 | 24.3 | _ |
| gi\|348580667 | PREDICTED: la-related protein 4-like | **0.652** | 0.675 | 1.7 | _ |
| gi\|348579652 | PREDICTED: aldo-keto reductase family 1 member B10-like | **0.539** | 0.834 | 7.6 | multicellular organismal process |
| *gi\|348571860 | PREDICTED: probable polyprenol reductase-like | **0.582** | **0.532** | 2.1 | glycoprotein metabolic process |
| gi\|348552472 | PREDICTED: LOW QUALITY PROTEIN: spectrin beta chain, brain 3-like | **0.51** | 0.826 | 0.3 | response to external stimulus |
| gi\|348580932 | PREDICTED: protein canopy homolog 2-like | **0.551** | 0.864 | 17.6 | _ |
| gi\|348561626 | PREDICTED: astrocytic phosphoprotein PEA-15-like | **0.47** | 0.755 | 7.7 | response to stimulus |
| gi\|348557903 | PREDICTED: heterogeneous nuclear ribonucleoprotein G-like | **0.504** | 0.829 | 9.7 | biological regulation |
| gi\|348567467 | PREDICTED: TATA-binding protein-associated factor 2N-like | **0.604** | 0.913 | 4 | _ |
| gi\|348586742 | PREDICTED: far upstream element-binding protein 1-like | **0.643** | 1.024 | 10.3 | RNA metabolic process |
| gi\|348571915 | PREDICTED: cytochrome c oxidase subunit 5B, mitochondrial-like | **0.374** | 0.67 | 9.3 | multicellular organismal process |
| gi\|348559730 | PREDICTED: 40S ribosomal protein S12-like | **0.498** | 0.675 | 6.1 | anatomical structure development |
| gi\|348550324 | PREDICTED: LOW QUALITY PROTEIN: scaffold attachment factor B1-like | **0.533** | 0.874 | 3.5 | biological regulation |
| gi\|348579929 | PREDICTED: signal recognition particle 14 kDa protein-like | **0.657** | 0.871 | 12.7 | response to stimulus |
| gi\|348572574 | PREDICTED: V-type proton ATPase subunit d 1-like | 0.898 | **0.664** | 8.5 | camera-type eye development |
| gi\|348566387 | PREDICTED: calcineurin subunit B type 1-like | 0.379 | **0.639** | 12.5 | angiogenesis |
| gi\|348550473 | ***PREDICTED: LOW QUALITY PROTEIN: ATP-binding cassette sub-family F member 1-like*** | 0.728 | **0.604** | 5.5 | defense response |
| gi\|348575898 | PREDICTED: lactoylglutathione lyase-like | 0.848 | **0.648** | 5.4 | regulation of programmed cell death |
| gi\|348554890 | ***PREDICTED: histone H2B type 1-C/E/G-like*** | 0.933 | **0.574** | 33.3 | defense response |
| gi\|348576314 | PREDICTED: serine/arginine-rich splicing factor 3-like | 0.648 | **0.568** | 18.3 | cellular macromolecular complex subunit organization |
| gi\|348578368 | PREDICTED: phosphoglycerate kinase 2-like | 0.752 | **0.588** | 7.9 | phosphate metabolic process |
| gi\|348587160 | ***PREDICTED: LOW QUALITY PROTEIN: phospholipase A-2-activating protein-like*** | 0.908 | **0.587** | 2.6 | defense response |
| gi\|348569476 | PREDICTED: histone H1.0-like | 0.6 | **0.293** | 11.3 | chromatin organization |
| gi\|348578235 | PREDICTED: cysteine and glycine-rich protein 1-like isoform 1 | 0.709 | **0.642** | 20.7 | cellular component organization |
| gi\|348578077 | PREDICTED: laminin subunit beta-3-like | 0.978 | **0.573** | 1.4 | cellular component organization |
| gi\|348569398 | PREDICTED: serine-threonine kinase receptor-associated protein-like | 1.142 | **0.601** | 14.6 | regulation of transcription |
| gi\|348583683 | PREDICTED: ubiquitin carboxyl-terminal hydrolase isozyme L3-like | 0.775 | **0.426** | 11.6 | response to insulin stimulus |
| gi\|348585397 | PREDICTED: 39S ribosomal protein L28, mitochondrial-like | 0.67 | **0.663** | 13.8 | gene expression |
| gi\|348580059 | PREDICTED: 40S ribosomal protein S17-like | 0.721 | **0.588** | 24.4 | homeostasis of number of cells |
| gi\|348564553 | PREDICTED: ragulator complex protein LAMTOR3-like | 0.772 | **0.645** | 8.5 | response to organic substance |
| gi\|348583768 | PREDICTED: propionyl-CoA carboxylase alpha chain, mitochondrial-like isoform 1 | 0.753 | **0.466** | 2.1 | lipid catabolic process |
| gi\|348574319 | PREDICTED: protein MEMO1-like | 1.096 | **0.559** | 8.8 | _ |
| gi\|348570813 | PREDICTED: heterochromatin protein 1-binding protein 3-like | 0.767 | **0.639** | 8.5 | _ |
| gi\|348556227 | PREDICTED: phosphatidylinositol-5-phosphate 4-kinase type-2 alpha-like, partial | 0.74 | **0.495** | 2.5 | organophosphate metabolic process |
| gi\|348577689 | PREDICTED: NADH dehydrogenase [ubiquinone] 1 alpha subcomplex subunit 10, mitochondrial-like | 0.953 | **0.492** | 6.5 | response to chemical stimulus |
| gi\|348588889 | PREDICTED: cytoplasmic dynein 1 light intermediate chain 1-like | 0.781 | **0.598** | 9.5 | microtubule-based process |
|  |  |  |  |  |  |
| Cluster 3: Up-regulated at day 1 and down-regulated at day 3 post-infection (8) | |  |  |  |  |
|  | |  |  |  |  |
| gi\|290543571 | ***neutrophil cationic peptide 1 type B preproprotein*** | **7.984** | 0.716 | 9.7 | defense response |
| gi\|348583804 | PREDICTED: multidrug resistance-associated protein 4-like isoform 1 | **2.003** | 0.624 | 1.1 | response to stimulus |
| *gi\|348586417 | ***PREDICTED: protein S100-A9-like*** | **2.744** | **0.637** | 9.2 | defense response |
| gi\|348553018 | PREDICTED: palmitoyl-protein thioesterase 1-like | **1.632** | 0.608 | 4.9 | response to stimulus |
| gi\|348572484 | PREDICTED: methionine aminopeptidase 1-like | 1.22 | **0.539** | 3 | peptidyl-amino acid modification |
| gi\|348550391 | PREDICTED: LOW QUALITY PROTEIN: DAZ-associated protein 1-like | 1.31 | **0.658** | 7.6 | gamete generation |
| gi\|348556450 | PREDICTED: long-chain-fatty-acid--CoA ligase 3-like | 1.249 | **0.596** | 7.6 | response to organic substance |
| gi\|348560062 | PREDICTED: LOW QUALITY PROTEIN: transcriptional activator protein Pur-beta-like | 1.206 | **0.645** | 8.1 | regulation of transcription |
|  |  |  |  |  |  |
| Cluster 4: Down-regulated at day 1 and up-regulated at day 3 post-infection (10) | |  |  |  |  |
|  | |  |  |  |  |
| gi\|348560174 | PREDICTED: platelet endothelial cell adhesion molecule-like | 0.714 | **1.65** | 2.5 | leukocyte migration |
| *gi\|348565171 | PREDICTED: src substrate cortactin-like isoform 1 | **0.621** | **1.835** | 12.2 | endocytosis |
| *gi\|348565223 | PREDICTED: cathepsin L1-like | **0.598** | **2.367** | 3.6 | gamete generation |
| gi\|348563941 | PREDICTED: dynein light chain roadblock-type 1-like | **0.384** | 1.272 | 29.2 | response to stimulus |
| gi\|348560331 | PREDICTED: myosin light chain 4-like | **0.315** | 1.214 | 23.6 | striated muscle contraction |
| gi\|348575686 | PREDICTED: cyclin-dependent kinase 1-like | **0.516** | 4593.74 | 5.8 | response to external stimulus |
| gi\|348557616 | ***PREDICTED: poliovirus receptor-related protein 2 isoform 1*** | **0.463** | 1.274 | 6.4 | defense response |
| gi\|348572554 | PREDICTED: enhancer of mRNA-decapping protein 4-like isoform 1 | **0.607** | 4593.74 | 2.3 | biopolymer catabolic process |
| gi\|348556794 | PREDICTED: HSPB1-associated protein 1-like | **0.529** | 1.253 | 1.1 | response to stimulus |
| gi\|348556319 | PREDICTED: hypothetical protein LOC100734603 | **0.471** | 1.514 | 5.9 | oxidation reduction |

Bold letters indicate the ratio of differential expression protein at day 1 or 3 dpi (Fold difference ≥1.5 or ≤0.67, P value <0.05). Asterisks indicate the common differential expression protein at both 1 and 3 dpi.
